# The Activation of the LIMK/Cofilin Signaling Pathway via Extracellular Matrix–Integrin Interactions Is Critical for the Generation of Mature and Vascularized Cardiac Organoids

**DOI:** 10.3390/cells12162029

**Published:** 2023-08-09

**Authors:** Ji-Min Noh, Seung-Cheol Choi, Myeong-Hwa Song, Kyung Seob Kim, Seongmin Jun, Jae Hyoung Park, Ju Hyeon Kim, Kyoungmi Kim, Tae Hee Ko, Jong-Il Choi, Jeong-An Gim, Jong-Hoon Kim, Yongjun Jang, Yongdoo Park, Ji Eun Na, Im Joo Rhyu, Do-Sun Lim

**Affiliations:** 1 Department of Cardiology, Cardiovascular Center, College of Medicine, Korea University, 73, Goryeodae-ro, Seongbuk-gu, Seoul 02841, Republic of Korea; wlals5344@gmail.com (J.-M.N.); choisc86@gmail.com (S.-C.C.); songmh616@gmail.com (M.-H.S.); ssks30101@gmail.com (K.S.K.); ellesejun@gmail.com (S.J.); jhpark3992@naver.com (J.H.P.); jhnkim86@gmail.com (J.H.K.); 2 R&D Center for Companion Diagnostic, SOL Bio Corporation, Suite 510, 27, Seongsui-ro7-gil, Seongdong-gu, Seoul 04780, Republic of Korea; 3 Department of Physiology, College of Medicine, Korea University, 73, Goryeodae-ro, Seongbuk-gu, Seoul 02841, Republic of Korea; kim0912@korea.ac.kr; 4 Division of Cardiology, Department of Internal Medicine, Anam Hospital, College of Medicine, Korea University, 73, Goryeodae-ro, Seongbuk-gu, Seoul 02841, Republic of Korea; kotiti2000@hanmail.net (T.H.K.); jongilchoi@korea.ac.kr (J.-I.C.); 5 Medical Science Research Center, Korea University Guro Hospital, 148, Gurodong-ro, Guro-gu, Seoul 08308, Republic of Korea; vitastar@korea.ac.kr; 6 Laboratory of Stem Cells and Tissue Regeneration, Department of Biotechnology, College of Life Sciences and Biotechnology, Korea University, Seoul 02841, Republic of Korea; jhkim@korea.ac.kr; 7 Department of Biomedical Sciences, College of Medicine, Korea University, 145 Anam-ro, Seongbuk-gu, Seoul 02841, Republic of Korea; jyj727@korea.ac.kr (Y.J.); ydpark67@korea.ac.kr (Y.P.); 8 Department of Anatomy College of Medicine, Korea University, 73, Goryeodae-ro, Seongbuk-gu, Seoul 02841, Republic of Korea; nje00@hanmail.net (J.E.N.); irhyu@korea.ac.kr (I.J.R.)

**Keywords:** cardiac organoids, mature cardiac organoids, vascularized cardiac organoids, LIMK/Cofilin signaling

## Abstract

The generation of mature and vascularized human pluripotent stem cell-derived cardiac organoids (hPSC-COs) is necessary to ensure the validity of drug screening and disease modeling. This study investigates the effects of cellular aggregate (CA) stemness and self-organization on the generation of mature and vascularized hPSC-COs and elucidates the mechanisms underlying cardiac organoid (CO) maturation and vascularization. COs derived from 2-day-old CAs with high stemness (H-COs) and COs derived from 5-day-old CAs with low stemness (L-COs) were generated in a self-organized microenvironment via Wnt signaling induction. This study finds that H-COs exhibit ventricular, structural, metabolic, and functional cardiomyocyte maturation and vessel networks consisting of endothelial cells, smooth muscle cells, pericytes, and basement membranes compared to L-COs. Transcriptional profiling shows the upregulation of genes associated with cardiac maturation and vessel formation in H-COs compared with the genes in L-COs. Through experiments with LIMK inhibitors, the activation of ROCK-LIMK-pCofilin via ECM–integrin interactions leads to cardiomyocyte maturation and vessel formation in H-COs. Furthermore, the LIMK/Cofilin signaling pathway induces TGFβ/NODAL and PDGF pathway activation for the maturation and vascularization of H-COs. The study demonstrates for the first time that LIMK/Cofilin axis activation plays an important role in the generation of mature and vascularized COs.

## 1. Introduction

Human pluripotent stem cell-derived cardiac organoids (hPSC-COs) generated in three-dimensional (3D) culture systems have been extensively used in studies of cardiac development, drug screening, and disease modeling [1]. To ensure the validity of drug testing and disease modeling, the use of mature hPSC-COs is necessary. Several strategies have recently been developed to enhance the maturation of hPSC-COs that are structurally and functionally similar to the heart using biochemical, electrical, and mechanical stimuli [2,3,4]. We also reported that the LEFTY-PITX2 signaling pathway plays important roles in cardiomyocyte (CM) maturation and ventricular-like CM subtype specification during the generation of cardiac mesoderm cell-derived COs [5]. However, current CO technologies are still immature, and little is known about the genes and mechanisms involved in CO maturation.

The formation of a functional vascular network that promotes oxygen and nutrient distribution in hPSC-COs to assess long-term drug toxicity is also crucial. To date, vascularized hPSC-COs resembling blood vessels have been generated using different strategies [2,3,4,6]. However, further studies into the functionality of vascularized COs will be necessary, particularly to elucidate the mechanisms underlying CO vascularization.

The Wnt signaling pathway is a key regulator of cardiac progenitor/stem cell self-renewal, differentiation, and cardiac morphogenesis [7,8]. One key element to generating robust and efficient CMs via Wnt signaling is the stemness of the hPSCs, which likely express uniform stem cell markers before the induction of Wnt signaling. When generating COs, cellular aggregate (CA) size is an important parameter in hPSC fate specification via the differential expression of noncanonical Wnt pathway genes [9,10,11]. However, prolonged incubations to form CAs can induce the spontaneous differentiation of CAs, resulting in a low-stemness CA state. In addition, self-organization is also a key developmental process during heart development that extends from the generation of cardiovascular progenitors to their specification [12,13].

Therefore, we hypothesized that CA stemness and self-organization are crucial for generating mature and vascularized COs via Wnt signal induction. To address this hypothesis, we investigated the ability of early high-stemness CAs and late low-stemness CAs to form mature CMs and vessels in self-organized COs generated via Wnt signaling without the administration of endothelial cells (ECs), ECM components, or exogenous angiogenic factors in hPSCs. Furthermore, we elucidated the genes and regulatory mechanisms involved in the formation of mature and vascularized COs.

## 2. Materials and Methods

### 2.1. Formation of COs from hPSCs

The H9 human embryonic stem cell (hESC) line was obtained from the WiCell Research Institute (Madison, WI, USA), and the TMOi001-A episomal human-induced PSC (hiPSC) line derived from CD34+ cord blood was purchased from Thermo Fisher Scientific (A18945, Waltham, MA, USA). The hPSCs were maintained in an E8 medium (Thermo Fisher Scientific) on plates coated with Matrigel (BD Biosciences, San Jose, CA, USA). For the formulation of hPSC-COs, poly-2-hydroxyethyl methacrylate (Poly-HEMA, P3932, Sigma-Aldrich, Saint Louis, MO, USA) was dissolved in cell-culture-tested ethanol (64-17-5, Merck Millipore) at 12 mg/mL and incubated at 40 °C overnight. The hPSCs were dissociated into single cells with the use of Accutase (Sigma-Aldrich) and then seeded onto a poly-HEMA-coated plate to form CAs at 1 × 10^6^ cells/cm^2^ in E8 supplemented with 2 μM of thiazovivin for 24 h. On day 0, the CAs were treated with 6 µM CHIR99021 (Sigma-Aldrich), a GSK3β inhibitor, in an RPMI 1640 + B27 minus insulin medium (RPMI/B27-Insulin, Thermo Fisher Scientific) for 48 h. On day 2, the CAs were treated with 2 µM IWP2 (Tocris Bioscience, Ellisville, MO, USA), a Wnt inhibitor, in an RPMI/B27–insulin medium for 48 h. On days 4 and 5, the medium was changed to an RPMI1640/B27–insulin medium. On day 7, the medium was changed to an RPMI 1640 + B27 minus vitamin A medium (RPMI/B27-Vitamin A, Thermo Fisher Scientific). Thereafter, the medium was changed every 2 days. The hPSC-COs were passed through strainers (BD Falcon, Franklin Lakes, NJ, USA) with 100 and 200 µm openings to maintain a uniform size, and hPSC-COs between 100 and 200 μm in diameter were used under pipette pressure on days 7 and 11. Phase contrast images were obtained using a DMI3000 B microscope (DMI 3000B, Leica Microsystems, Wetzlar, Germany).

### 2.2. Quantitative Reverse Transcription Polymerase Chain Reaction (qRT-PCR)

Using a TRIzol reagent (TR-118, MRC Inc., Cincinnati, OH, USA), total RNA was extracted from the hPSC-COs in accordance with the manufacturer’s protocol. The total RNA concentration was measured using a NanoDrop spectrophotometer (ND-1000, Thermo Fisher Scientific), and 500 ng of total RNA was used for complementary DNA synthesis via supplementation with an M-MLV reverse transcriptase (28025-013, Invitrogen, Carlsbad, CA, USA) in a volume of 20 μL at 37 °C for 50 min. The qRT-PCR was performed using an SYBR Green Mixture (170-8880, Bio-Rad Laboratories, Hercules, CA, USA), and the results were recorded using an MYiQ2 detection system (Bio-Rad Laboratories). Relative gene expression levels were quantified on the basis of Ct and normalized to the reference gene, glyceraldehyde 3-phosphate dehydrogenase (*GAPDH*). Intron spanning primers were designed using ProbeFinder (https://www.roche-applied-science.com accessed on 1 August 2022) to avoid genomic DNA amplification. The primer sequences used for the qRT-PCR are listed in Table S1.

### 2.3. Immunofluorescence Staining

The hPSC-COs were washed twice with phosphate-buffered saline (PBS) and fixed with 2% paraformaldehyde (Sigma-Aldrich) dissolved in PBS for 30 min. The fixed hPSC-COs were permeabilized with 0.1% Triton X-100 in PBS for 30 min, washed in PBS + 0.1% Tween 20 (PBST), and blocked with 5% normal goat serum (NGS, Thermo Fisher Scientific) in PBST for 2 h. The hPSCs were then stained with primary antibodies (Table S2). The primary antibodies were incubated with 5% NGS in PBST at 4 °C overnight. The hPSC-COs were washed thrice in PBST and incubated with the following secondary antibodies at room temperature (RT) for 1 h: Alexa Fluor 488 goat anti-mouse IgG1 (1:1000; A21121, Invitrogen), Alexa Fluor 488 chicken anti-rabbit IgG1 (1:1000; A21441, Invitrogen), Alexa Fluor 594 goat anti-mouse IgG1 (1:1000; A11005, Invitrogen), Alexa Fluor 594 goat anti-rabbit IgG1 (1:1000; A11012, Invitrogen), and Alexa 647 goat anti-rabbit IgG (1:1000; A21244, Invitrogen). Mitochondrial activity was measured using the cell-permanent mitochondrion-selective dye Mitotracker (M7511, 100 nM, Invitrogen) in hESC-derived COs (hESC-COs). The nuclei were stained with 4′,6-diamidino-2-phenylindole (DAPI, D9542, Sigma–Aldrich), and the stained cells were mounted using a fluorescent mounting solution (S3023, DAKO, Carpinteria, CA, USA). Immunofluorescence images were acquired using a confocal fluorescence microscope (LSM800, Carl Zeiss, Oberkochen, Germany).

### 2.4. Western Blotting

The hESC-COs were washed twice with PBS and lysed with a 1× cell lysis buffer (9803, Cell Signaling Technology, Danvers, MA, USA) plus 1 mM of phenylmethylsulfonyl fluoride (P7626, Sigma–Aldrich). Then, the protein concentrations of the samples were determined using a Bradford assay dye reagent (500-0006, Bio-Rad Laboratories). The sample protein (10 μg) was boiled in 1× loading dye for 8 min, electrophoresed on a 10% sodium dodecyl sulfate–polyacrylamide gel, and transferred to a polyvinylidene fluoride membrane (10600023, Thermo Fisher Scientific). The membranes were blocked with 5% bovine serum albumin (A0100-010, GenDEPOT) containing 1× TBST (a mixture of Tris-buffered saline and Tween 20; WH400028806, 3M) at RT for 1 h, incubated with the corresponding antibodies (Table S3), washed thrice with TBST, and further incubated with a horseradish peroxidase-conjugated secondary antibody (1:4000; Cell Signaling Technology) at RT for 1 h. Chemiluminescence was visualized using ECL Plus reagents (32132, Thermo Fisher Scientific) and a ChemiDoc™ Touch Imaging System (1708370, Bio-Rad Laboratories). Protein levels were normalized to GAPDH (G8795, Sigma-Aldrich).

### 2.5. Transmission Electron Microscopy (TEM)

The hESC-COs were fixed with 2% paraformaldehyde and 2.5% glutaraldehyde in a 0.1 M phosphate buffer (pH 7.4) at 4 °C overnight. The samples were postfixed in 1% osmium tetroxide, dehydrated, and embedded in Eponate-12 resin (Ted Pella). Then, 1 μm section blocks were obtained using a Reichert-Jung Ultracut E ultramicrotome (Leica Microsystems, Wetzlar, Germany), stained with toluidine blue, and imaged using a Carl Zeiss Axio microscope. Afterward, 60 nm sections were collected from each block and stained with uranyl acetate/lead citrate. The images were obtained via TEM (H-7500, Hitachi, Tokyo, Japan) at 80 kV and analyzed using Image J (version 1.50i).

### 2.6. Beating Analysis Using Captured Videos

The contractile properties of the hESC-COs were determined by analyzing videos obtained via a microscope (Nikon, Ti2-E, Tokyo, Japan) at 10× magnification. The videos were captured at 50 frames per second and analyzed using NIS software (Nikon), which counts variations in light intensity in a selected region for 25 s. The beating kinetics of each sample were estimated to identify beating regularity and homogeneity.

### 2.7. Whole-Cell Patch Clamp Recordings

Action potential (AP) was measured during whole-cell configuration, and the electrophysiological phenotypes in the hESC-COs were determined. Patch-clamp experiments were conducted using an Axopatch 200B amplifier (Axon Instrument) at RT. The hESC-COs were placed in a chamber mounted on an inverted microscope and continuously superfused with a normal Tyrode solution containing the following: 143 mM of NaCl, 5.4 mM of KCl, 5 mM of HEPES, 0.33 mM of NaH_2_PO_4_, 5.5 mM of glucose, 1.8 mM of CaCl_2_, and 0.5 mM of MgCl_2_ (pH 7.4). The patch pipette solution was composed of the following: 140 mM of KCl, 5 mM of EGTA, 5 mM of glucose, 5 mM of HEPES, 5 mM of Mg-ATP, and 1 mM of MgCl_2_ (pH 7.2). The AP recording and analysis collected data according to all the details previously described [5].

### 2.8. Ca^2+^ Transient Analysis

For a Ca^2+^ transient analysis, whole H-COs and L-COs were loaded with 4 μg/mL of Fluo-4 AM (F14201, Invitrogen) in a fresh medium at 37 °C for 45 min. Ca^2+^ imaging was performed using a confocal fluorescence microscope (LSM800, Carl Zeiss).

### 2.9. Image Rendering

The images were reconstructed into 3D shapes by using ZEN software (CarlZeiss Microscopy GmbH, Jena, Germany). The acquired confocal Z-stacks, comprising up to 10 images, were reconstructed into 3D images. Any increase in brightness was applied uniformly across an entire z-projected image.

### 2.10. RNA Sequencing (RNA-Seq) Analysis

The hESC-COs were collected on days 5, 15, and 25 of differentiation for an RNA-Seq analysis. The total RNA was extracted using a Trizol reagent (TR-118, MRC) in accordance with the manufacturer’s instructions. Concentrations were determined according to previous research methods [5]. Libraries wfor the RNA-Seq analysis were prepared using a QuantSeq Library Prep kit (Lexogen, Inc., Vienna, Austria). High-throughput sequencing was performed using a NextSeq 500 (Illumina, Inc., San Diego, CA, USA). Differentially expressed genes (DEGs) were analyzed using ExDEGA software (Biogen, Inc., Seoul, Republic of Korea). Gene classification was based on searches performed using the DAVID (http://david.abcc.ncifcrf.gov/ accessed on 9 May 2023) and Medline databases (http://www.ncbi.nlm.nih.gov/ accessed on 9 May 2023), gene ontology (GO, https://www.ebi.ac.uk/QuickGO/ accessed on 9 May 2023), R studio (https://rstudio.com/ accessed on 9 May 2023), and STRING (https://cytoscape.org/ accessed on 9 May 2023). Heatmaps were generated using R studio.

### 2.11. Pharmacologic Reagents

A potent LIM kinase inhibitor, LIMKi3 (BMS-5, SYN-1024, SYNkinase), was prepared by suspending it in DMSO, and DMSO alone was used as a control. The sample was treated with H-COs on day 11 of differentiation with 10 μM of LIMKi3 or an equal volume of DMSO. After 2 days, the medium was changed, and the sample was analyzed via qRT-PCR and Western blot on day 15 of differentiation.

### 2.12. Statistical Analysis

All statistical values were expressed as means ± standard deviations (SDs). Significant differences between means were determined using Student’s *t*-test or an ANOVA followed by the Student–Newman–Keuls test. Statistical significance was set at *p* < 0.05. Data were analyzed using Prism version 8.0.2 (GraphPad, San Diego, CA, USA).

## 3. Results

### 3.1. CAs Formed for 2 Days Generate More Ventricular-like and Atrial-like CMs Than Those Formed for 5 Days

To address the effects of CA stemness and self-organization on cardiac differentiation and maturation in hESC-COs, we compared CAs which were formed for 2 days and highly expressed stemness markers with CAs which were formed for 5 days and were partially differentiated into three germ layers, as shown via qRT-PCR and immunostaining. We labeled the COs as follows: H-COs, COs generated from the 2-day-old CAs with high stemness, and L-COs, COs generated from the 5-day-old CAs with low stemness.

**Figure 1:**
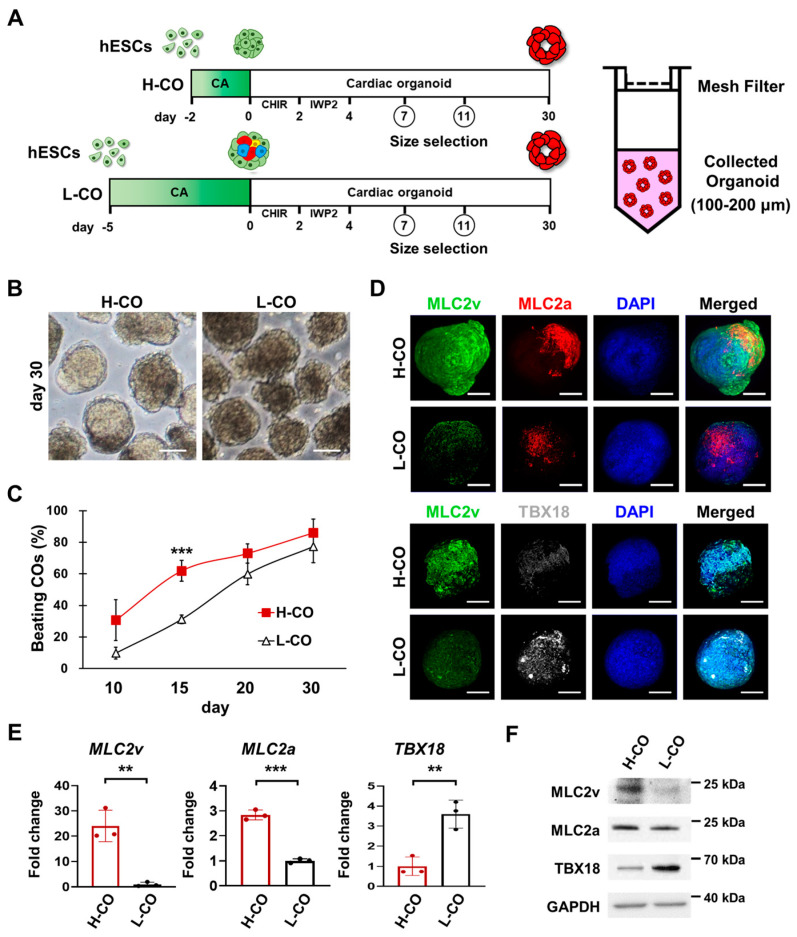
Longer incubation time for CA formation correlates with an increase in spontaneous differentiation and a decrease in cardiac differentiation upon cardiac induction in H9 hESCs. (**A**) Schematic diagram depicting the protocol used to generate the H-COs and L-COs. (**B**) Phase contrast images of H-COs and L-COs on day 30. Scale bars = 100 μm. (**C**) The estimated average percentages of beating H-COs and L-COs on days 10, 15, 20, and 30 of differentiation. *n* = 25. *** *p* < 0.001. (**D**) Immunofluorescence images of a ventricular marker (MLC2v; green), an atrial marker (MLC2a; red), and a nodal marker (TBX18; white) in H-COs and L-COs on day 30 of differentiation. Nuclei were stained with 4′,6-diamidino-2-phenylindole (DAPI; blue). Scale bars = 50 μm. (**E**) qRT-PCR and (**F**) Western blot analyses of MLC2v, MLC2a, and TBX18 in H-COs and L-COs on day 30 of differentiation. *n* = 3. ** *p* < 0.01. *** *p* < 0.001.

To generate H-COs and L-COs, we used 1 × 10^6^ cells and treated them with a Wnt signaling activator (CHIR) and a Wnt signal inhibitor (IWP; Figure 1A, Figures S1 and S2). On days 7 and 11, H-COs and L-COs in sizes ranging from 100 μm to 200 μm were selected using cell strainers and cultured until day 30 (Figure 1A,B and Figure S3). The average percentages of beating H-COs and L-COs were investigated from days 10, 15, 20, and 30 of differentiation. The percentages of beating H-COs and L-COs increased on these days. On days 10 and 15 of differentiation, we observed that the percentage of beating H-COs was higher than that of the L-COs (31% vs. 9.8% and 61.9% vs. 31.3%). However, the average percentages of beating H-COs and L-COs were similar on days 20 (72.9% vs. 59.9%) and 30 (86.0% vs. 77.3%) of differentiation (Figure 1C).

We used CM subtype markers to carry out a qRT-PCR, Western blot, and immunostaining to investigate the differentiation of the H-COs and L-COs into CM subtypes. MLC2v and MLC2a were more highly expressed in the H-COs than in the L-COs. However, the level of TBX18 expression was lower in the H-COs than in the L-COs on day 30 (Figure 1D–F).

### 3.2. Structural and Metabolic Maturation Are Increased in H-COs Compared with L-COs

**Figure 2:**
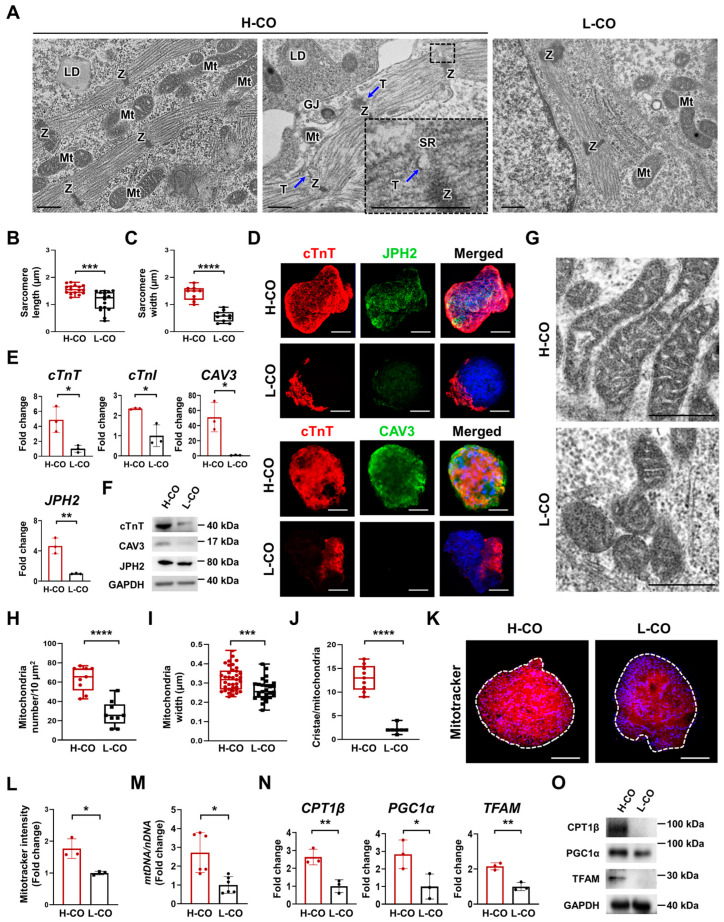
Highly organized sarcomeres and well-developed t-tubules are found in H-COs, and their level of metabolic maturity is higher than L-COs derived from H9 hESCs. (**A**) Representative TEM images of H-COs and L-COs. Coupled t-tubules and terminal cisternae on the sarcoplasmic reticulum are visible in H-COs. Black dashed lines indicate a magnified view of t-tubule formation in the H-COs. GJ: gap junction, LD: lipid droplet, Mt: mitochondria, SR: sarcoplasmic reticulum, T: t-tubule, Z: Z-line. Scale bars = 0.5 μm. Quantification of sarcomere (**B**) length and (**C**) width in H-COs and L-COs. *n* = 16. *** *p* < 0.001. **** *p* < 0.0001. (**D**) Immunofluorescence images of cTnT (red), JPH2 (green), and CAV3 (green) in H-COs and L-COs. Nuclei were stained with DAPI (blue). Scale bars = 50 μm. (**E**) qRT-PCR analysis of a total CM marker (cTnT), a mature CM marker (cTnI), and t-tubule markers (CAV3 and JPH2) in H-COs and L-COs. *n* = 3. * *p* < 0.05. ** *p* < 0.01. (**F**) Representative Western blotting images of cTnT, CAV3, and JPH2 expression in H-COs and L-COs. Glyceraldehyde 3-phosphate dehydrogenase (GAPDH) was used as an endogenous control. (**G**) TEM images showing mitochondria in H-COs and L-COs. Scale bars = 0.5 μm. (**H**) Quantification of mitochondrial numbers in H-COs and L-COs. *n* = 9. *** *p* < 0.001. **** *p* < 0.0001. (**I**) Quantification of mitochondrial width in H-COs (*n* = 37) and L-COs (*n* = 27). (**J**) Quantification of cristae per mitochondrion in H-COs (*n* = 9) and L-COs (*n* = 7). (**K**) Immunofluorescence images of an active mitochondrion-specific fluorescent dye, MitoTracker (red), and DAPI (blue) in H-COs and L-COs. White dashed lines indicate the boundaries of H-COs and L-COs. Scale bars = 50 μm. (**L**) Quantification of the MitoTracker stain in H-COs and L-COs. *n* = 3. * *p* < 0.05. (**M**) Relative mtDNA of the copy number in H-COs and L-COs. *n* = 6. * *p* < 0.05. (**N**) qRT-PCR analysis of metabolism markers (CPT1β, PGC1α, and TFAM) in H-COs and L-COs. *n* = 3. * *p* < 0.05. ** *p* < 0.01. (**O**) Representative Western blotting images of CPT1β, PGC1α, and TFAM in H-COs and L-COs. GAPDH was used as an endogenous control.

To assess the structural maturity of the hESC-COs, we examined the ultrastructures of the sarcomeres and mitochondria in the H-COs and L-COs via TEM on day 30 of differentiation. The H-COs had more organized sarcomeres, which were defined by distinct Z-lines and closely aligned with the mitochondria, than the L-COs. The t-tubule formation responsible for heart contraction and ion channel signaling, which is an indicator of cardiac maturity, was observed in the H-COs (Figure 2A). In addition, the average length (1.6 μm vs. 1.1 μm) and width (1.4 μm vs. 0.6 μm) of sarcomeres were higher in the H-COs than in the L-COs (Figure 2B,C). Immunostaining on day 30 revealed that CAV3+ and JPH2+ CMs were more evenly and abundantly found in the H-COs than in the L-COs (Figure 2D). The mRNA and protein expression levels of *cTnT*, *cTnI*, *CAV3*, and *JPH2* were markedly higher in the H-COs than in the L-COs (Figure 2E,F).

We also investigated the maturity of the mitochondria, which regulate metabolic energy. TEM images showed that the mitochondria were large and elongated in the H-COs, whereas they were small and round in the L-COs. The mitochondrial cristae were denser and more uniform in the H-COs than in the L-COs (Figure 2G). The number (63.7 vs. 27.2) and width (0.32 μm vs. 0.27 μm) of mitochondria were significantly higher in the H-COs than in the L-COs (Figure 2H,I). The number of mitochondrial cristae (10.9 vs. 2.14) was also higher in the H-COs than in the L-COs (Figure 2J). The mitochondrial activity detected via immunofluorescence staining with MitoTracker, an active mitochondria-specific fluorescent dye, was higher in the H-COs than in the L-COs (Figure 2K,I). To quantify the mitochondrial content of the COs, we analyzed the ratio of mitochondrial DNA (mtDNA) and nuclear DNA (nDNA) via a qRT-PCR. We found that the ratio of mtDNA/nDNA was significantly increased in the H-COs compared with in the L-COs (Figure 2M). The mRNA and protein expression levels of *CPT1β*, *PGC1α*, and a regulator of fatty acid activity (*TFAM*) were markedly higher in the H-COs than in the L-COs (Figure 2N,O). These results suggested that the metabolic activity of the mitochondria was more mature in the H-COs than in the L-COs. The H-COs had a significantly higher proportion of organoids (48.8% vs. 23.6%) with cavity formation than the L-COs on day 30 (Figure S4). 

### 3.3. Beating and Electrophysiological Properties of H-COs Indicate Ventricular-like CMs

**Figure 3:**
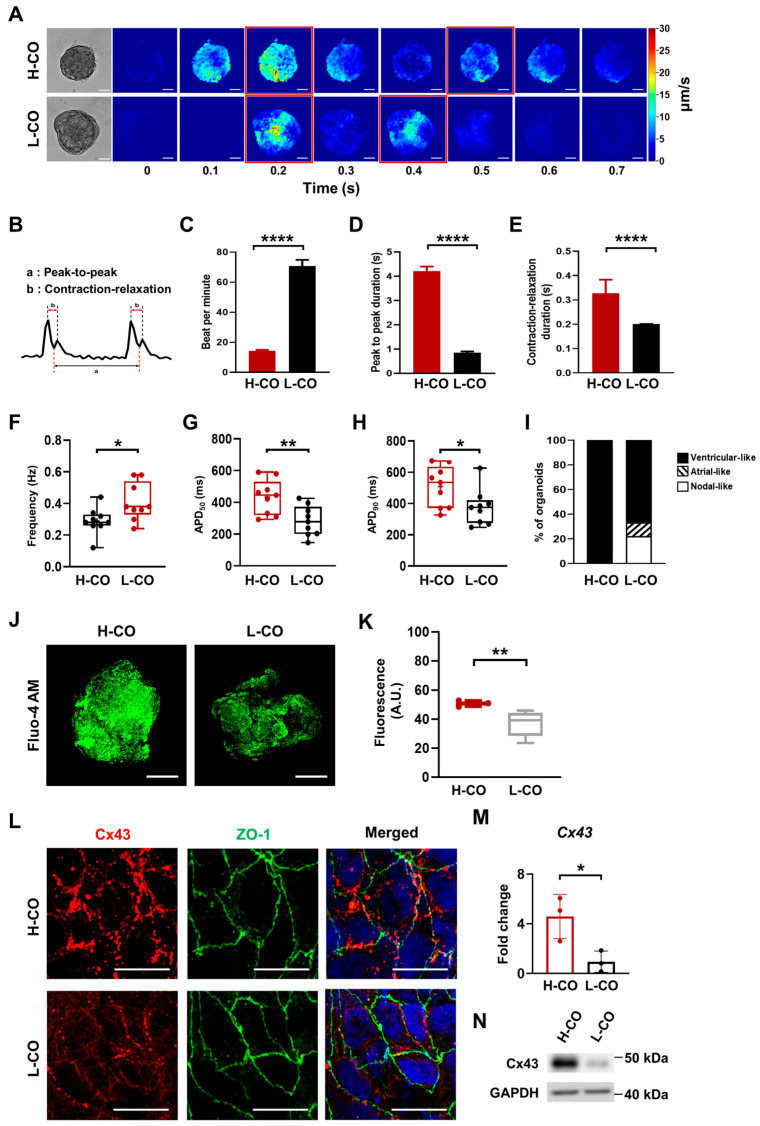
Electrophysiological and contractile properties indicate ventricular-like CMs in H-COs but not L-COs from H9 hESCs. (**A**) Morphologies and magnitudes representative of motion velocities of H-COs and L-COs. Motion velocity shows contraction and relaxation, and the red box indicates each peak point. Scale bars = 20 μm. (**B**) Diagram of the beat profile corresponding to the contraction and relaxation of CMs. (**C**–**E**) Comparisons of beats per minute, peak-to-peak durations, and contraction–relaxation durations in H-COs and L-COs. *n* = 5. **** *p* < 0.0001. (**F**) Beating frequency was analyzed by counting spontaneous APs per minute. * *p* < 0.05. (**G**,**H**) APD50 and APD90 in H-COs and L-COs. *n* = 9. * *p* < 0.05. ** *p* < 0.01. (**I**) Percentile distribution of the three types of APs in H-COs and L-COs. *n* = 9. (**J**) Ca^2+^ fluorescent images of H-COs and L-COs under a confocal microscope with Fluo-4 AM. Scale bars = 50 μm. (**K**) Fluorescence intensity of Fluo-4 AM in H-COs and L-COs was measured using Image J. *n* = 5. ** *p* < 0.01. (**L**) Immunofluorescence images of a gap junction protein, Cx43 (red), and a tight junction protein, ZO-1 (green), in H-COs and L-COs. Nuclei were stained with DAPI (blue). Scale bars = 10 μm. (**M**) qRT-PCR and (**N**) Western blot analyses of Cx43 expression in H-COs and L-COs. *n* = 3. * *p* < 0.05.

To investigate CM subtype specification and functional maturation, we examined the beating properties of the H-COs and L-COs by analyzing videos taken under a microscope on day 30 of differentiation. The morphologies and magnitudes representative of the motion velocities of the H-COs and L-COs are shown in Figure 3A and Video S1. The H-COs showed a large and slow beat pattern, whereas the L-COs exhibited a small and fast beat pattern. On day 30, synchronized beating occurred in different regions in the H-COs, unlike in the L-COs derived from the H9 hESCs (Figure S5). A diagram of the beat profile corresponding to the contraction and relaxation of the CMs is provided in Figure 3B. The number of beats per minute in the H-COs was low, whereas the peak-to-peak duration and contraction–relaxation duration were significantly higher in the H-COs than in the L-COs (Figure 3C–E). These results demonstrated that unlike the L-COs, the H-COs, which have synchronized beating, fewer beats per minute, and longer peak-to-peak and contraction–relaxation durations, have the electrophysiological properties and beating characteristics of ventricular-like CMs.

The electrophysiological properties of the H-COs and L-COs were recorded via a whole-cell patch-clamp analysis. Representative images of ventricular-, atrial-, and nodal-type CMs are shown in Figure S6A. The H-COs had a reduced beating frequency (0.29 Hz vs. 0.4 Hz) compared with the L-COs (Figure 3F and Figure S7), and the H-COs showed prolonged APD_50_ (434.5 ms vs. 283.4 ms) and APD_90_ (509.3 ms vs. 375.6 ms) times compared with the L-COs (Figure 3G,H and Figure S6B). All the H-COs showed ventricular-like CMs, whereas only 66.7% of the L-COs contained ventricular-like CMs; of the remaining L-COs, 11.1% and 22.2% had atrial- and nodal-like CMs, respectively (Figure 3I). We measured Ca^2+^ transients in the H-COs and L-COs by using the Ca^2+^ indicator Fluo-4 AM to assess the functional maturation of the CMs (Figure 3J). We found that the Fluo-4 AM fluorescence intensity was significantly higher in the H-COs than in the L-COs, indicating that the H-COs were more functionally mature than the L-COs (Figure 3K).

### 3.4. Junctional Structures between CMs Are Better Aligned in H-COs Than in L-COs

The cardiac intercalated disc that connects adjacent CMs is composed of a gap junction, an adherens junction, and a desmosome [14]. We used TEM to examine the connections between CMs through the intercalated discs of the H-COs and L-COs. On day 30, we found more organized intercalated discs containing adherens junctions, gap junctions, and desmosomes in the H-COs than in the L-COs (Figures S7 and S8). Immunofluorescence staining showed that Cx43, a gap junction marker, was evenly expressed at the junctions between cells in the H-COs, whereas it was only partially distributed in the L-COs. The ZO-1 tight junction protein was evenly expressed in both the H-COs and L-COs (Figure 3I). The mRNA and protein expression levels of *Cx43* were higher in the H-COs than in the L-COs (Figure 3M,N). These results suggested that the intercalated discs in the H-COs were better organized than those in the L-COs. Collectively, these results show that CA stemness and self-organization are critical for the structural and functional maturation of COs.

### 3.5. Formation of a Capillary Network with a Lumen Is Predominantly Found in H-COs

**Figure 4:**
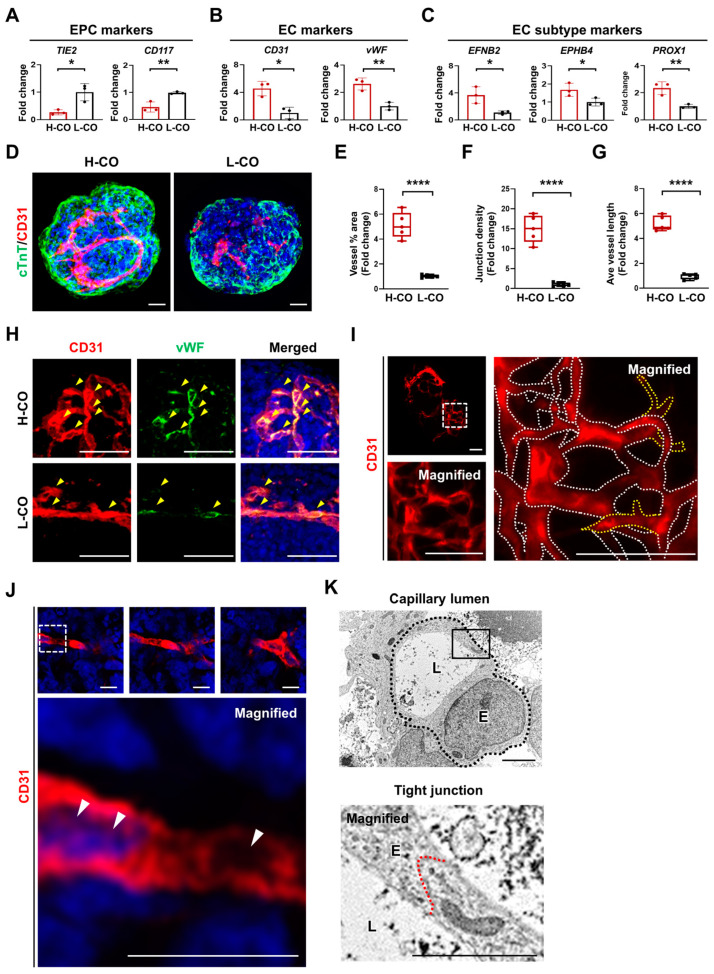
More ECs, EC subtypes, and capillary network formation with lumens are found in H-COs than in L-COs derived from H9 hESCs. qRT-PCR analysis of (**A**) EPC markers (TIE2 and CD117), (**B**) EC markers (CD31 and vWF), and (**C**) EC subtype markers (EFNB2, EPHB4, and PROX1) in H-COs and L-COs. *n* = 3. * *p* < 0.05. ** *p* < 0.01. (**D**) Immunofluorescence images of a total CM marker (cTnT; green) and an EC marker (CD31; red) in H-COs and L-COs. Scale bars = 20 μm. Quantification of (**E**) vessel percentage area, (**F**) junction density, and (**G**) average vessel length in H-COs and L-COs. Data were analyzed by quantifying CD31+ levels in five whole organoids. **** *p* < 0.0001. (**H**) Immunofluorescence images of CD31 (red) and vWF (green) in H-COs and L-COs. Yellow arrowheads indicate cells co-expressing CD31 and vWF. Scale bars = 20 μm. (**I**) Immunofluorescence images showing a vessel structure with a clear lumen (dashed white lines) and CD31+ endothelial tip cells (dashed yellow line) in H-COs. Scale bars = 20 μm. (**J**) Immunofluorescence images showing the formation of a lumen consisting of CD31+ ECs in H-COs. White arrowheads indicate the vascular lumen. Scale bars = 0.05 μm. (**K**) TEM images showing the formation of a vascular lumen (L), ECs (E), and tight junctions (thick red lines) in H-COs. Scale bars = 1 μm.

On day 30, we examined the expression of ECs and fibroblasts other than CMs that constitute the heart. We specifically investigated the expression of endothelial progenitor cell (EPC) markers, EC markers, and EC subtype markers via a qRT-PCR. Interestingly, *TIE2* and *CD117* were more strongly expressed in the L-COs than in the H-COs (Figure 4A), whereas the expression levels of *CD31*, *vWF*, and the EC subtype markers, *EFNB2*, *EPHB4*, and *PROX1* were all significantly higher in the H-COs than in the L-COs on day 30 (Figure 4B,C). Conversely, immunostaining and the qRT-PCR analysis revealed that *VIMENTIN* and *FSP1* did not differ significantly between the H-COs and L-COs, and FSP1+ cells were similarly observed in both the H-COs and L-COs (Figure S9). We further examined EC expression via immunostaining. In cTnT+/CD31+ hESC-COs, ECs were found more abundantly in the H-COs than in the L-COs (Figure 4D and Video S2). The vessel area (5.14-fold), junction density (14.9-fold), and average vessel length (5.2-fold) were significantly upregulated in the H-COs compared with those in the L-COs (Figure 4E–G). Most of the CD31+ H-COs co-expressed vWF, whereas only few of the CD31+ L-COs co-expressed vWF (Figure 4H). We next investigated whether the H-COs formed a capillary network and lumen. Confocal imaging showed the formation of a complex and interconnected network of CD31+ endothelial tubes and terminal sprout-like structures (Figure 4I). Confocal images (Figure 4J) and a video clip (Video S3) revealed the formation of a lumen made from CD31+ ECs in the H-COs. TEM images also displayed the formation of a lumen and typical tight junctions between ECs in the H-COs, and the capillary and thickness diameters were 8.46 and 0.52 μm, respectively (Figure 4K). These results demonstrated that differentiation into ECs and EC subtypes was highly induced in the H-COs, which formed a capillary network with the lumen.

### 3.6. Mature Vessels Covered by Pericytes, SMCs, and a BM Are Formed in H-COs

**Figure 5:**
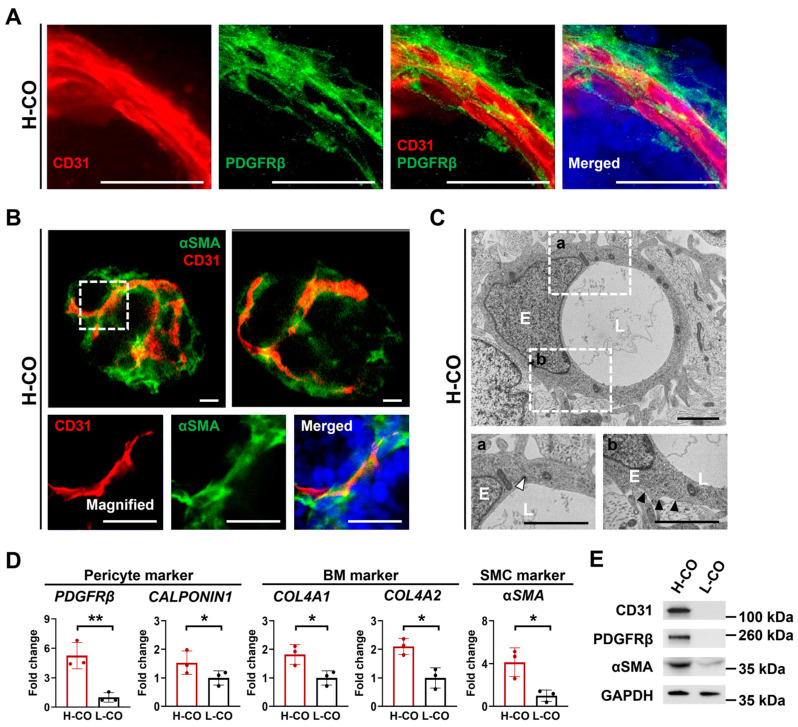
Mature vessels covered by pericytes, SMCs, and vascular BM are found in H-COs derived from H9 hESCs. Immunofluorescence images showing the CD31+ endothelial lumen covered by (**A**) PDGFRβ+ pericytes and (**B**) αSMA + SMCs. Nuclei were stained with DAPI (blue). Scale bars = 20 µm. (**C**) Representative TEM images showing the formation of ECs (E), vascular lumens (L), tight junctions (white arrowheads), and BM (black arrowheads) in H-COs. The dotted boxes are enlarged in the lower panels. Scale bars = 1 μm. (**D**) qRT-PCR analysis of pericyte markers (PDGFRβ and CALPONIN), an SMC marker (αSMA), and BM markers (COL4A1 and COL4A2) in H-COs and L-COs. *n* = 3. * *p* < 0.05. ** *p* < 0.01. (**E**) Representative Western blotting images of CD31, PDGFRβ, and αSMA expression in H-COs and L-COs. GAPDH was used as an endogenous control.

We next examined whether mature vessels covered by pericytes, SMCs, and a BM were generated in the H-COs on day 30. Confocal images revealed CD31+ endothelial lumens covered by PDGFRβ and αSMA, indicating that pericytes and SMCs were being assembled into the vessel wall, as typically found in human blood vessels (Figure 5A,B and Figure S10). The TEM analysis also confirmed the formation of a BM and typical tight junctions between ECs in the H-COs (Figure 5C). The quantitative analysis of vascularization-related gene expression revealed that the expression levels of *PDDGFRβ*, *CALPONIN1*, *αSMA*, *COL4A1* and *COL4A2* were significantly higher in the H-COs than in the L-COs (Figure 5D). Furthermore, Western blotting confirmed the strong expression of CD31, PDGFRβ, and αSMA proteins in the H-COs (Figure 5E). These results showed that the H-COs generated mature vessels covered by pericytes, SMCs, and a BM, indicating that CAs with high stemness and self-organization are critical for the generation of vascularized COs.

### 3.7. Transcriptional Profiling Reveals CM Maturation and Vessel Formation in H-COs at the Molecular Level

**Figure 6:**
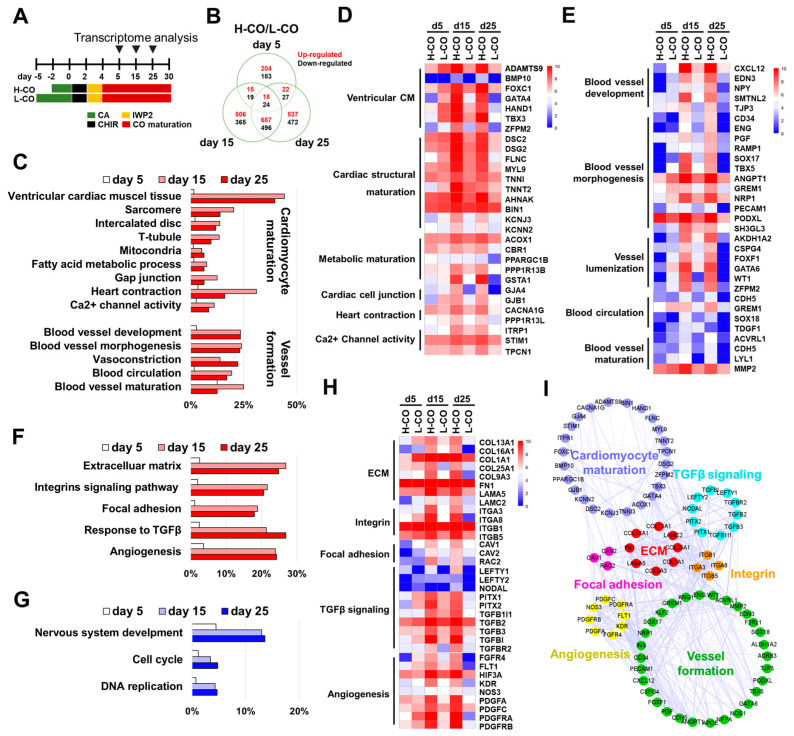
Transcriptome profiling indicates that the genes involved in CM maturation and vessel formation are upregulated in H-COs compared with those in L-COs from H9 hESCs. (**A**) Schematic of the transcriptome analysis of H-COs and L-COs on days 5, 15, and 25. (**B**) Venn diagram depicting the number of genes upregulated (red) and downregulated (black) > 2-fold between H-COs and L-COs on days 5, 15, and 25. (**C**) GO analysis of genes associated with CM maturation and vessel formation upregulated in H-COs compared with L-COs on days 5 (white), 15 (pink), and 25 (red). Clustered heatmaps of DEGs involved in (**D**) CM maturation and (**E**) vessel formation in H-COs and L-COs on days 5, 15, and 25. Pathway analysis of genes (**F**) upregulated in H-COs compared with L-COs on days 5 (white), 15 (pink), and 25 (red), and (**G**) downregulated in H-COs compared with L-COs on days 5 (white), 15 (sky blue), and 25 (blue). (**H**) Clustered heatmap of DEGs involved in ECM, integrin, focal adhesion, TGFβ signaling, and angiogenesis signaling in H-COs and L-COs on days 5, 15, and 25. (**I**) Network analysis of DEGs associated with CM maturation and vessel formation in H-COs compared with those in L-COs. Circles and lines represent genes and protein interactions between genes, respectively.

To elucidate the genes and signaling pathways underlying the differences in CM maturation and vessel formation between the H-COs and L-COs, we analyzed the transcriptomes of the H-COs and L-COs on days 5, 15, and 25 (Figure 6A). Among the 3573 differentially expressed genes, 204, 506, and 537 were upregulated by more than 2-fold in the H-COs compared with those in the L-COs on days 5, 15, and 25, respectively, whereas 183, 365, and 472 genes were downregulated in the H-COs compared with those in the L-COs on days 5, 15, and 25, respectively (Figure 6B; Table S4).

A heatmap analysis revealed that heart-specific genes were upregulated in the H-COs and L-COs on days 5, 15, and 25, but other organ-specific genes, such as those for the brain, kidney, liver, lung, and skin, were expressed at considerably lower levels (Figure S12). DEGs with more than 2-fold changes in expression between the H-COs and L-COs were subjected to GO pathway analyses to evaluate the biological consequences of the changes in gene expression (Figure 6C,F,G; Tables S5 and S6).

Clustered GO enrichments revealed that the genes involved in CM maturation and vessel formation were upregulated in the H-COs compared with the L-COs on days 5, 15, and 25 (Figure 6C; Table S5). However, the expression levels of genes associated with vessel formation did not change on day 5 compared with days 15 and 25 (Figure 6C,F,G). Most DEGs in the H-COs and the L-COs on days 5, 15, and 25 are compared in the heatmaps in Figure 6D,E. Our heatmap analysis revealed that the expression levels of genes associated with CM maturation (i.e., CM maturation, ventricular CMs, cardiac structural maturation, metabolic maturation, cardiac cell junctions, heart conduction, and Ca^2+^ channel activity), and vessel formation (i.e., blood vessel development, blood vessel morphogenesis, vessel lumenization, blood circulation, and blood vessel maturation) were higher in the H-COs than in the L-COs on days 15 and 25 (Figure 6D,E).

The GO pathways of the major upregulated genes (ECM, integrin signaling pathway, focal adhesion, response to TGFβ, and angiogenesis) and downregulated genes (nervous system development, cell cycle, and DNA replication) in the H-COs are compared with those in the L-COs in Figure 6F. The heatmaps revealed that the DEGs were mostly changed in the H-COs compared with those in the L-COs on days 15 and 25. The genes associated with ECM (*COL13A1*, *COL16A1*, *COL1A1*, *COL25A1 COL9A3*, *FN1*, *LAMA5*, and *LAMC2*), integrin (*ITGA3*, *ITGA8*, *ITGAB1*, and *ITGA5*) and focal adhesion signaling (*CAV1*, *CAV2*, and *RAC2*) were more highly expressed in the H-COs than in the L-COs on days 15 and 25. Interestingly, the genes encoding TGFβ signaling (*LEFTY1*, *LEFTY2*, *NODAL*, *PITX1*, *PITX2*, *TGFB1I1*, *TGFB2*, *TGFB3*, *TGFBI*, and *TGFR2*) were more highly expressed in the H-COs than in the L-COs on days 15 and 25. Strikingly, the genes encoding angiogenesis (*FGFR4*, *FLT1* [*VEGFR1*], *HIF3A*, *KDR* [*VEGFR2*], *NOS3* [*eNOS*], *PDGFA*, *PDGFC*, *PDGFRA* [*PDGFRα*], and *PDGFRB* [*PDGFRβ*]) were also enriched in the H-COs compared with the L-COs on days 15 and 25 (Figure 6H). A protein network analysis via Cytoscape revealed the interactions among the DEGs involved in the ECM, integrin, focal adhesion, TGFβ signaling, and angiogenesis in the H-COs on day 25. Furthermore, molecular networks shared between CM maturation and vessel formation participated in the ECM, integrin, focal adhesion, and TGFβ signaling pathways (Figure 6I). Therefore, our transcriptional profiling showed that the genes and pathways involved in CM maturation and vessel formation were highly activated in the H-COs.

### 3.8. Activation of ROCK-LIMK-pCofilin, LEFTY-NODAL, pVEGFR, pPDGFR, and peNOS Pathways via ECM–Integrin Interactions Led to CM Maturation and Vessel Formation in COs

**Figure 7:**
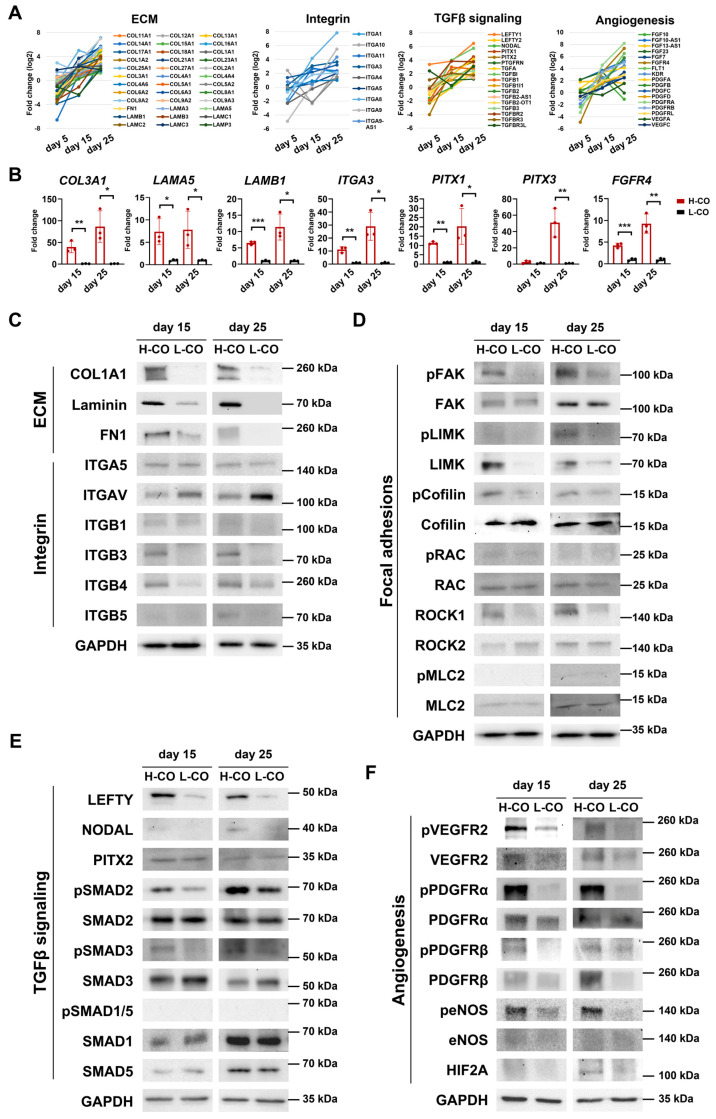
Putative signaling pathways underlying CM maturation and vessel formation in COs generated aggregates with pluripotency (H-COs) via Wnt induction from H9 hESCs. (**A**) Gene expression levels of ECM, integrin, TGFβ signaling, and angiogenesis in RNA-Seq data from H-COs and L-COs on days 5, 15, and 25. (**B**) qRT-PCR analysis of ECM genes (COL3A1, LAMA5, and LAMB1), an integrin gene (ITGA3), TGFβ signaling pathway genes (PITX1 and PITX3), and an angiogenesis gene (FGFR4) in H-COs and L-COs on days 15 and 25. *n* = 3. * *p* < 0.05. ** *p* < 0.01. *** *p* < 0.001. Representative Western blotting images of (**C**) ECM proteins (COL1A1, Laminin A, and FN1), and integrin molecules (ITGA5, ITGAV, ITGB1, ITGB4, and ITGB5); (**D**) focal adhesion signaling proteins (pFAK, FAK pLIMK, LIMK, pCofilin, Cofilin, pRAC, RAC, ROCK1, ROCK2, pMLC2, and MLC2); (**E**) TGFβ signaling pathway proteins (LEFTY, NODAL, PITX2, pSMAD2, SMAD2, pSMAD3, pSMAD1/5, SMAD1, and SMAD5); and (**F**) angiogenesis proteins (pVEGFR2, VEGFR2, PDGFRα, pPDGFRα, PDGFRβ, pPDGFRβ, eNOS, peNOS, and HIF2A) in H-COs and L-COs on days 15 and 25.

Because our transcriptional profiling analysis identified the ECM–integrin, focal adhesion, TGFβ signaling, and angiogenesis signaling pathways as potential mechanisms underlying CM maturation and vessel formation in H-COs, we further validated these signaling pathways via Western blot analyses. The expression levels (>log 2-fold change) of the ECM, integrin, the TGFβ signaling pathway, and angiogenesis genes were upregulated in the RNA-Seq results in the H-COs compared with the L-COs on days 15 and 25 (Figure 7A). A qRT-PCR validated that among the genes selected via RNA-Seq analysis, the ECM genes (*COL3A1*, *LAMA5*, and *LAMB1*), an integrin gene (*ITGA3*), the TGFβ signaling pathway genes (*PITX1* and *PITX3*), and an angiogenesis gene (*FGFR4*) were upregulated in the H-COs compared with the L-COs on days 15 and 25 (Figure 7B).

Integrins are heterodimeric cell surface receptors that participate in multiple critical cellular processes, including adhesion, ECM organization, signaling, survival, and proliferation [15]. The ECM–integrin–cytoskeleton linkage mediates mechano-transduction signaling in cardiac microenvironment regulation [16]. In our study, ECM proteins (COL1A, Laminin, and FN1) were markedly upregulated, with a moderate increase in ITGB1 and significant increases in TGB3 and ITGB4 in the H-COs compared to the L-COs on days 15 and 25 (Figure 7C). Conversely, ITGAV strongly decreased in the H-COs compared with the L-COs on days 15 and 25. These results indicate that interactions between integrin subtypes and specific ECM proteins (COL1A, Laminin A, and FN1) could regulate the downstream focal adhesion signaling involved in generating mature H-COs via the Wnt induction of hPSC aggregates.

A Rho-associated protein kinase, ROCK1, was strongly upregulated in the H-COs compared with the L-COs on days 15 and 25. pFAK and total LIMK1 markedly increased in the H-COs compared with the L-COs on days 15 and 25, whereas pLIMK was moderately increased in the H-COs compared with the L-COs only on day 25. pCofilin was markedly increased in the H-COs compared with the L-COs on days 15 and 25, but pRAC and pMLC did not differ between the H-COs and L-COs on either day. These results suggested that the activation of the ROCK1-pFAK-pLIMK-pCofilin pathways could play critical roles in the ECM–integrin-triggered maturation of H-COs (Figure 7D).

LEFTY and NODAL signals belong to the TGFβ superfamily and are known to regulate left–right asymmetry in the heart development of mammals [17]. LEFTY was strikingly upregulated, and NODAL was slightly upregulated in the H-COs compared with the L-COs on days 15 and 25. However, PITX2, a downstream effector in the left–right signal pathway, did not differ between the H-COs and L-COs on day 15 or 25. As expected, the phosphorylation of SMAD2 and SMAD3 was enhanced in the H-COs compared with the L-COs on days 15 and 25, but pSMAD1/5 was not detected in the H-COs or L-COs (Figure 7E).

The signaling pathway genes involved in angiogenesis or vessel formation and identified via the transcriptional profiling analysis were then validated via Western blotting. The phosphorylation of PDGFRα and VEGFR2 was strongly upregulated in the H-COs compared to the L-COs on days 15 and 25. The total PDGFRα, PDGFRβ, and VEGFR2 protein levels were also moderately increased in the H-COs compared with the L-COs on days 15 and 25. Interestingly, peNOS was dramatically enhanced in the H-COs compared with the L-COs on days 15 and 25; conversely, the HIF2A protein was moderately increased in the H-COs compared with the L-COs only on day 25 (Figure 7F). Collectively, the ECM–integrin, ROCK-pFAK-pLIMK-pCofilin, LEFTY-NODAL, pPDGFRα, pPDGFRβ, pVEGFR, and peNOS signaling pathways were likely involved in CM maturation and vessel formation in COs.

### 3.9. LIMK/Cofilin Signaling Pathways Play Critical Roles in CM Maturation and Vessel Formation in H-COs

**Figure 8:**
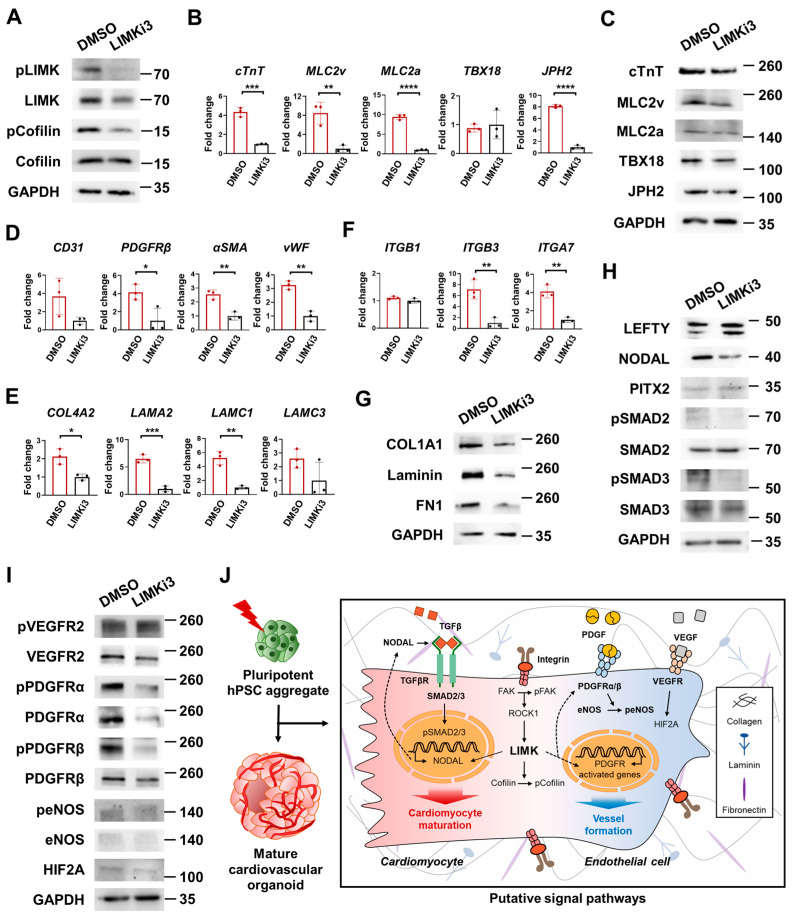
LIMK/Cofilin signaling pathways are critical for CM maturation and vessel formation during H-COs generation. (**A**) Western blotting images of LIMK/Cofilin signaling pathway (LIMK, pLIMK, Cofilin, and pCofilin) in H-COs treated with DMSO and LIMKi3. (**B**) Representative qRT-PCR analysis and (**C**) Western blotting images of cardiomyocyte maturation marker proteins (cTnT, MLC2v, MLC2a, TBX18, and JPH2) in H-COs treated with DMSO and LIMKi3. *n* = 3. ** *p* < 0.01. *** *p* < 0.001. **** *p* < 0.0001. (**D**) Representative qRT-PCR analysis of vessel formation markers (CD31, PDGFRβ, αSMA, and vWF) in H-COs treated with DMSO and LIMKi3. *n* = 3. * *p* < 0.05. ** *p* < 0.01. (**E**) qRT-PCR of ECM signaling pathway genes (COL4A2, LAMA2, LAMC1, and LAMC3) and (**F**) integrin genes (ITGB1, ITGB3 and ITGA7) in H-COs treated with DMSO and LIMKi3. *n* = 3. * *p* < 0.05. ** *p* < 0.01. *** *p* < 0.001. Western blot analysis of (**G**) ECM genes (COL1A, Laminin, and FN1), (**H**) TGFβ signaling genes (LEFTY, NODAL, PITX2, SMAD2, pSMAD2, SMAD3, and pSMAD3) and (**I**) angiogenesis genes (VEGFR2, pVEGFR2, PDGFRα, pPDGFRα, PDGFRβ, pPDGFβ, eNOS, peNOS, and HIF2A) in H-COs treated with DMSO and LIMKi3. (**J**) Schematic showing activation of LIMK/Cofilin signaling pathway via ECM-integrin interaction is critical for generation of mature and vascularized cardiac organoids derived from cell aggregates with high stemness.

Because the LIMK/Cofilin signaling pathway genes were significantly activated in CM maturation and in the vascularized H-COs compared with the L-COs, we defined the role of LIMK/Cofilin signaling in CM maturation and vessel formation. We treated H-COs with DMSO and LIMKi3, a potent LIM kinase inhibitor, on day 11, and analyzed them via a qRT-PCR and Western blot on day 15. We found that the protein expression levels of the total LIMK, pLIMK and pCofilin were significantly downregulated in the H-COs treated with LIMKi3 compared with the H-COs treated with DMSO control (Figure 8A). Next, we investigated whether LIMK inhibition affected CM maturation and vessel formation in the H-COs. The gene (Figure 8B) and protein (Figure 8C) expression levels of the CM maturation-associated genes *cTnT*, *MLC2v*, *MLC2a*, and *JPH2* decreased in the LIMKi3-treated H-COs. In addition, the gene expression levels of the vessel formation-related genes *PDGFRβ*, *αSMA*, and *vWF* significantly decreased in the LIMKi3-treated H-COs compared with the DMSO control-treated H-COs (Figure 8D).

We investigated whether the ECM–integrin interaction, LEFTY-NODAL, pPDGFRα, pPDGFRβ, pVEGFR, and peNOS signaling pathways were affected in the H-COs treated with LIMKi3. The gene expression levels of *COL4A2*, *LAMA2*, *LAMC1*, *LAMC3*, *ITGB3*, and *ITGA7* significantly decreased in the LIMKi3-treated H-COs (Figure 8E,F). We used Western blotting to further validate the signal. The protein expression levels of ECM genes (COL1A1, Laminin, and FN1) decreased in the LIMKi-treated H-COs (Figure 8G). Although NODAL decreased in the LIMKi3-treated group, interestingly, the protein level of LEFTY increased in the LIMKi3-treated group compared with the DMSO control group. In addition, the phosphorylation of SMAD2 and SMAD3 increased in the LIMKi3-treated group (Figure 8H).

The total PDGFRα, pPDGFRα, and pPDGFRβ protein levels dramatically decreased in the LIMKi3-treated H-COs (Figure 8I). peNOS was slightly downregulated in the LIMKi3-treated group compared with the DMSO control group. However, the total expression levels of VEGFR2, pVEGFR2, and HIF2A did not differ between the LIMKi3-treated and DMSO control groups. Collectively, we demonstrated for the first time that LIMK/Cofilin signaling plays an important role in the generation of mature and vascularized COs with respect to ECM–integrin interactions, TGFβ signaling pathways (NODAL, pSMAD2 and pSMAD3), and the pathways of vessel formation (PDGFRα, pPDGFRα, pPDGFRβ, and peNOS).

## 4. Discussion

The heart develops from mesoderm progenitors, the first and second heart fields, which subsequently differentiate into CMs, ECs, and fibroblasts, forming the distinct anatomical structures of the heart [18]. In this study, we established a novel method for the production of mature and vascularized COs by mimicking in vivo heart development in hPSCs through a self-organization strategy, simulating cardiac development without requiring treatment with ECs or exogenous angiogenic factors (i.e., VEGF). H-COs with high stemness demonstrated predominant ventricular, structural, metabolic, functional, and CM maturation. Furthermore, their neovascularization increased, and their mature blood vessels covered by pericytes, SMCs, and a BM were greater in number than those in L-COs. Collectively, these results showed that CA stemness and self-organization are critical for the structural and function maturation of COs. Our findings indicate that the induction of Wnt signaling in CAs composed of undifferentiated OCT4+ hPSCs provided a microenvironment of ECM, heart component cells, and soluble factors similar to that involved in in vivo heart development.

LIMK is a serine/threonine-protein kinase that includes LIMK1 and LIMK2 members. It is an upstream protein that regulates Cofilin and catalyzes Cofilin phosphorylation. LIMK-mediated Cofilin phosphorylation is critically involved in various physiological and pathological processes [19]. LIMK induces cardioprotective signal activation by stimulating Cofilin in cardiomyocytes [20]. It also triggers actin filament stabilization in the cardiovascular system [21]. We hypothesized that the LIMK/Cofilin signaling pathway played an important role in CM maturation and vessel formation during CO generation. To block LIMK activity, we selected a specific LIMK inhibitor, LIMKi3 [22].

Mimicking the cardiac microenvironment, which is composed of the ECM, different cell types, and soluble factors, as closely as possible improves the functional maturation and structural organization of laboratory-grown 3D cardiac tissues [16]. Collagen type I supports the maturation of murine and hPSC-CMs [23,24,25], and collagen type III is a major structural component in blood vessels [26]. In addition, maturing neonate valves predominantly express collagens, including COL1A1 and COL3A1, in defined proximal and distal regions [27]. An ECM containing a complex mixture of laminin and fibronectin supports the early maturation of mouse ESC-derived CMs [28]. Laminins containing α4 and α5 chains are the major isoforms found in vessel walls [29]. Laminin 10, composed of laminins α5, β1, and γ1 chains, is detectable primarily in the endothelial BMs of capillaries and venules after birth [30]. Lee et al. [31] generated mature heart organoids from mESC-derived embryo bodies by using the laminin–entactin complex and fgf4 in a self-organizing manner. Interestingly, the gene expression levels of *COL4A2*, *LAMA2*, and *LAMC1* and the protein expression levels of COL1A1, Laminin, and FN1 significantly decreased in LIMKi3-treated H-COs (Figure 8E,G). 

Integrins are heterodimeric transmembrane receptors that are expressed in all cells, including those in the heart, and they participate in multiple critical cellular processes, including adhesion, ECM organization, signaling, survival, and proliferation [15]. The ECM–integrin–cytoskeleton linkage, via FAK activation, particularly mediates mechano-transduction signaling in regulating the cardiac microenvironment [15,16]. An impaired sarcomeric architecture and ventricular CMs are observed in integrin β1-deficient ESCs and mice [32]. Integrin α5 and β1 are induced in hPSC-CM maturation via the induction of the FAK activity [33]. A fibronectin and laminin (FN/LN) mixture promotes the efficient differentiation of CMs from the H7 and H9 hESC lines and causes an increase in the FN receptor integrin β5 (ITGB5), the LN receptor integrin β4 (ITGB4), pFAK, and p-ERKs compared with gelatin. Collagen type I, laminin, and integrin β1 are associated with cardiac differentiation [34] and the alteration of the cholinergic regulation of the L-type Ca^2+^ current in CMs [35] ITGB1 and ITGB3 regulate the formation and stability of blood vessels, exhibiting a distinct dynamic nanoscale organization inside focal adhesions [36,37]. A network-like pattern of LAMA4 and LAMA5, which correspond to the localization of the laminin-adhesion molecules ITGA6 and ITGB4, is observed in the blood-flow-induced remodeling of the BM [38]. In this study, we found that the gene expression levels of *ITGB3* and *ITBA7* were downregulated in LIMKi3-treated H-COs (Figure 8F). Our findings demonstrate that LIMK/Cofilin signaling interacted with ECM–integrin molecules during CO formation.

We previously demonstrated that the LEFTY-NODAL signaling pathway is important for maturation and ventricular cardiac organoid formation in cardiac mesoderm cells [5]. NODAL ligands bind to dimers of TGF-β type I and type II receptors [39]. NODAL is a ligand that activates the receptor complex to induce the phosphorylation of Smad2 or Smad3 and subsequent nuclear localization [40]. The NODAL signaling pathway is negatively regulated by LEFTY [41]. Our results showed that the protein levels of NODAL were downregulated in the LIMKi3-treated H-COs. In addition, the pSMAD2 and pSMAD3 levels were downregulated in the LIMKi3-treated group, demonstrating association with the TGFβ superfamily signaling pathway. However, the protein levels of LEFTY were upregulated in the LIMKi3-treated H-COs (Figure 8H). This demonstrated that a feedback loop of the NODAL/LEFTY system was formed after LIMK inhibitor treatment.

Many organoid systems are limited by their lack of functional vascular networks for oxygen and nutrient distribution that contribute to the maturation of adult-like organoids through paracrine signaling [42]. We found that VEGFR2, pVEGFR2, PDGFRα, PDGFRβ, pPDGFRα, peNOS, and HIF2A levels were enhanced in the H-COs when compared with those in the L-COs (Figure 8F). Vessel maturation requires mural cell recruitment, ECM generation, vessel wall specialization for structural support, and vessel function regulation [43]. The spatial localization and kinetic delivery of angiogenic signals, including VEGF, FGF2, and PDGF, in the ECM are crucial to the proper assembly and maturation of new vascular structures [44,45]. 

PDGFRβ (−/−) hearts fail to form dominant coronary vessels on the ventral heart surface, have a thin myocardium, and completely lack coronary vascular SMCs [43]. Pericyte recruitment to EC tubes during vasculogenic tube assembly and stabilization leads to the specific induction of the ECM and coincidently upregulates integrins, which bind to fibronectin, nidogens, laminin isoforms, and COL IV+ [43,46,47]. Similarly, CD31+ endothelial tubes in vascular organoids are covered by PDGFRβ+ pericytes and COL IV+ BMs [48]. HIF2α regulates angiogenic extracellular signaling [49] and promotes vessel remodeling [50]. eNOS phosphorylation is associated with increased microvascular permeability [51], myocardial vascular maturation, and angiogenesis [52]. Consistent with our results, previous findings revealed that the enhanced maturation of CMs is closely associated with increased neovascularization in COs, indicating a crosstalk between newly forming vessels and CMs [53]. Interestingly, the downregulation of total PDGFRα, pPDGFRα, and pPDGFRβ in the LIMKi3-treated H-COs indicated that they were closely related to the activation of the PDGFR pathway, suggesting that these genes and pathways played important roles during angiogenesis and vessel maturation in organoids. However, there was no change in the expression levels of the total VEGFR2 and pVEGFR2 proteins (Figure 8I). These results indicate that VEGFR2-mediated pathways independently regulate secretion through other target proteins. 

The conventional view is that the activity of LIMK/Cofilin stabilizes the actin cytoskeleton [54,55,56]. However, the contribution of axin remodeling to LIMK/Cofilin in the generation of mature and vascularized COs is currently unclear. It will be interesting to investigate whether and how alterations in actin remodeling induced by LIMK/Cofilin signaling and their association with mature and vascularized COs are involved in future studies.

In summary, we generated mature and vascularized COs called H-COs by triggering Wnt activation in hESCs and hiPSCs. In comparison with L-COs, the H-COs showed dominant ventricular, structural, metabolic, and functional CM maturation, increased vessel area, junction density, and vessel length, and matured vessels covered by pericytes, SMCs, and a BM with a lumen. Transcriptional profiling revealed the upregulation of genes associated with cardiac maturation and vessel formation in the H-COs compared with those in the L-COs. Here, we demonstrated for the first time that activation of ROCK-LIMK-pCofilin via ECM–integrin interactions leads to cardiomyocyte maturation and vessel formation in hPSC-COs. Furthermore, we demonstrated that the LIMK/Cofilin signaling pathway induces TGFβ/NODAL and PDGF pathway activation for the maturation and vascularization of hPSC-COs. We proved that CA stemness and the self-organizing process are critical for the maturation and vascularization of hPSC-COs. The mature and vascularized COs are an attractive tool for drug discovery and disease modeling in the cardiac field.

## Data Availability

Not applicable.

## Supplementary Materials

The following supporting information can be downloaded at: https://www.mdpi.com/article/10.3390/cells12162029/s1.
