# Supplementary material for: The Activation of the LIMK/Cofilin Signaling Pathway via Extracellular Matrix–Integrin Interactions Is Critical for the Generation of Mature and Vascularized Cardiac Organoids
Source: Cells. 2023 Aug 9;12(16):2029. doi: 10.3390/cells12162029 (PMC10453200; doi:10.3390/cells12162029)
Supplement: Supplementary file 1 [file cells-12-02029-s001.zip › Cells_Supplemental Material_Revision_Final.pdf]

Supplemental Material

Activation of LIMK/Cofilin signaling pathway via extracellular matrix-integrin interaction is critical for generation of mature and vascularized cardiac organoids

Supplementary Results

Longer incubation time during CA formation leads to spontaneous differentiation of hESCs

We first determined the optimal seeding density for CA formation. Different numbers of H9 hESCs (0.25, 0.5, 1, 2, 3, and 4 × 10^6) were seeded in each well of a Poly-HEMA coated 6-well culture plate. We found that a seeding density of 1 × 10^6 cells was optimal for CA formation (Figure S1). To investigate the spontaneous differentiation of CAs into three germ layers during their formation, we incubated cells on Poly-HEMA-coated 6-well culture plates from day 1 to day 5 under 3D culture conditions (Figure S2A). During that time, we observed changes in the CA morphology and increases in their diameters. The mean diameters of the CAs on days 1–5 was 32.6, 72.1, 102.2, 135.2, and 156.2 μm, respectively (Figure S2B and S2D).

To investigate the spontaneous differentiation of CAs over time, we examined the gene expression patterns of cell lineage markers on days 1–5 of differentiation. Pluripotency makers (*OCT4* and *NANOG*) were gradually downregulated over time and significantly downregulated on days 4 and 5. An ectodermal marker, *OTX2* mRNA, was significantly upregulated from days 3 to 5, and another ectodermal marker, *ZIC1* mRNA, was significantly upregulated on day 5. A mesoderm marker, *MESP1* mRNA, was significantly upregulated from day 4 to day 5, and

another mesoderm marker, *T* mRNA, was significantly upregulated on day 5. The *CXCR4* mRNA endoderm marker was significantly upregulated on days 4 and 5, and *AFP* mRNA was significantly upregulated on day 5 (Figure S2C).

We further examined CAs by immunostaining them with different cell lineage markers: a pluripotent marker (OCT4), an ectoderm marker (NESTIN), mesoderm markers (T, MIXL1 and VEGFR2), and an endoderm marker (FOXA2). Furthermore, the three germ layer markers were not expressed by the CAs on 2 days, and they were distinctly expressed on 5 days (Figure S2D). The average numbers of cells in the CAs on days 2 and 5, as estimated by counting DAPI-stained cells, were 166.6 and 418.1, respectively (Figure S2E and S2F). This result demonstrates that allowing CA formation to continue for longer incubation times induces spontaneous differentiation in a time-dependent manner in hESCs.

Generation of uniform-sized COs using mesh filters during CO formation derived from H9 hESCs

We used cell strainers with different mesh sizes to select hPSC-COs of a uniform size (Fig. 1a). On day 7, the average diameters of hPSC-COs < 100 μm, 100–200 μm, and > 200 μm were 80.1 μm, 152.4 μm, and 279.7 μm, respectively (Figure S3A and S3B). The average numbers of hESC-COs < 100 μm, 100–200 μm, and > 200 μm were 22.1, 42.0, and 6.8, respectively (Figure S3C). On day 30, the average percentages of hESC-COs < 100 μm, 100–200 μm, and > 200 μm that were beating were 27.5%, 65.0%, and 7.5%, respectively (Figure S3D).

CM maturation and vessel formation in H-CO compared to L-CO in hiPSCs at day 30

We investigated the differentiation and maturation of hiPSC-derived H-COs and L-COs into cellular components of the heart, such as CMs, ECs, SMCs, and fibroblasts. We examined the

proportions of MLC2v+, CD31+, and FSP-1+ cells in those H-COs and L-COs by immunostaining. MLCv+ CMs and CD31+ ECs were more abundantly detected in H-COs than L-COs, whereas FSP-1+ fibroblasts were similarly observed in both H-COs and L-COs (Figure S11A). We investigated the differentiation of CM subtypes in hiPSC-derived H-COs and L-COs using qRT-PCR. The expression of a ventricular CM marker (*MLC2v*) and an atrial CM marker (*MLC2a*) was significantly higher in the H-COs than the L-COs, whereas the expression of a nodal CM marker (*TBX18*) decreased significantly over time in both H-COs and L-COs (Figure S11B). We investigated the maturation of CMs in hiPSC-derived H-COs and L-COs using qRT-PCR to quantify the expression of a total CM marker (*cTnT*), a mature CM marker (*cTnI*), T-tubule markers (*CAV3* and *JPH2*), a metabolic marker (*CPT1β*), and a gap junction marker (*Cx43*). We found significantly more CM maturation genes in H-COs than in L-COs (Figure S11C-E). Next, we investigated marker genes for vessel formation in hiPSC-derived H-COs and L-COs using qRT-PCR. We found that the expressions of EC markers (*CD31* and *vWF*), a pericyte marker (*PDGFRβ*), an SMC marker (*αSMA*), and a BM marker (*COL4A1*) were all significantly higher in H-COs than in L-COs (Figure S11F), whereas the expression of a fibroblast marker (*FSP-1*) did not differ significantly between the H-COs and L-COs (Figure S11G). Thus, we have shown that the protocol we have established is applicable to hiPSCs as well as hESCs.

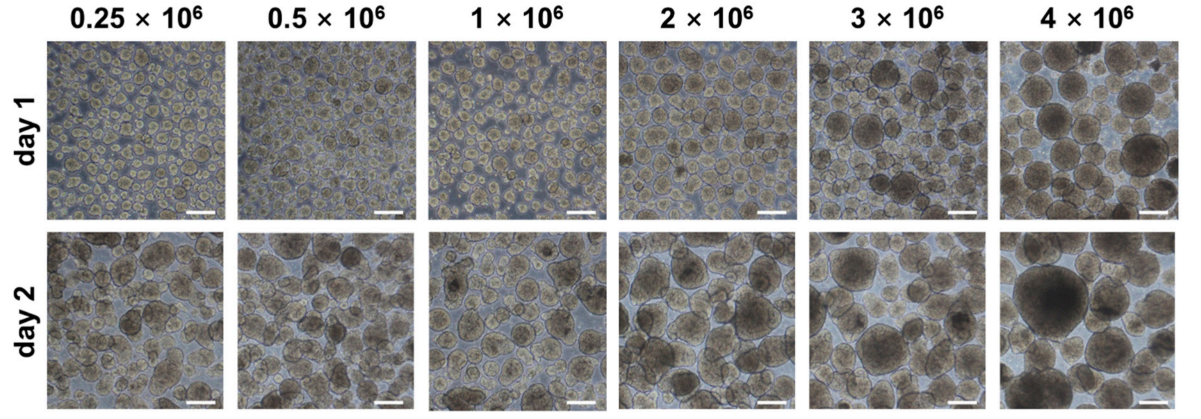

Figure S1. Optimization of cell seeding density to generate hESC-COs. Phase contrast images from days 1 and 2 showing the morphologies of CAs started with different seeding densities. Scale bars = 100 μm.

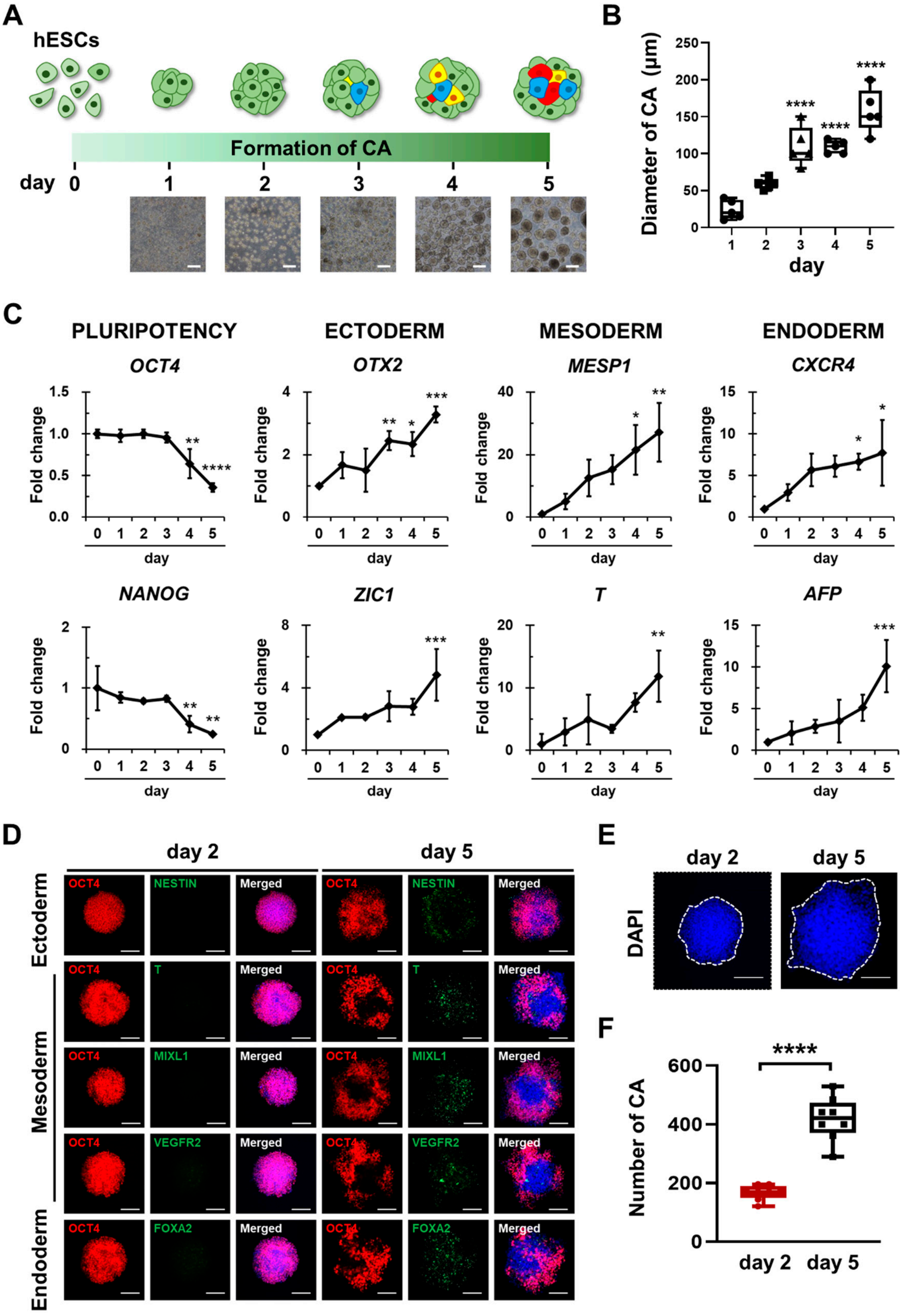
A
hESCs
Formation of CA
day 0 1 2 3 4 5
B
Diameter of CA (μm)
day
C
PLURIPOTENCY
ECTODERM
MESODERM
ENDODERM
OCT4
OTX2
MESP1
CXCR4
NANOG
ZIC1
T
AFP
Fold change
day
D
day 2
day 5
Ectoderm
Mesoderm
Endoderm
OCT4
NESTIN
T
MIXL1
VEGFR2
FOXA2
Merged
E
day 2
day 5
DAPI
F
Number of CA
day 2 day 5

Figure S2. Longer incubation time for CA formation increases spontaneous differentiation in H9 hESCs. (A) Schematic diagram showing a positive correlation between the incubation period and spontaneous differentiation in CAs from days 1 to 5. Scale bars = 100 µm. (B) Mean diameter of CAs on days 1–5. n = 5. (C) qRT-PCR analysis of pluripotency markers (*OCT4* and *NANOG*), ectoderm markers (*OTX2* and *ZIC1*), mesoderm markers (*MESP1* and *ISL1*), and endoderm markers (*CXCR4* and *FOXA2*) in undifferentiated hESCs on day 0 and in CAs on days 1–5. n = 3. *$p < 0.05$. **$p < 0.01$. ***$p < 0.001$. ****$p < 0.0001$. (D) Immunostaining of a pluripotent marker (OCT4), an ectoderm marker (NESTIN), mesoderm markers (T, MIXL1 and VEGFR2), and an endoderm marker (FOXA2) on days 2 and 5. Nuclei were stained with DAPI (blue). Scale bars = 50 µm. (E) Nuclei were stained with DAPI (blue) on days 2 and 5. White dashed lines indicate the CA boundaries. Scale bars = 50 µm. (F) Quantification of the average number of cells per aggregate in CAs formed after 2 and 5 days of incubation. n = 8. ****$p < 0.0001$.

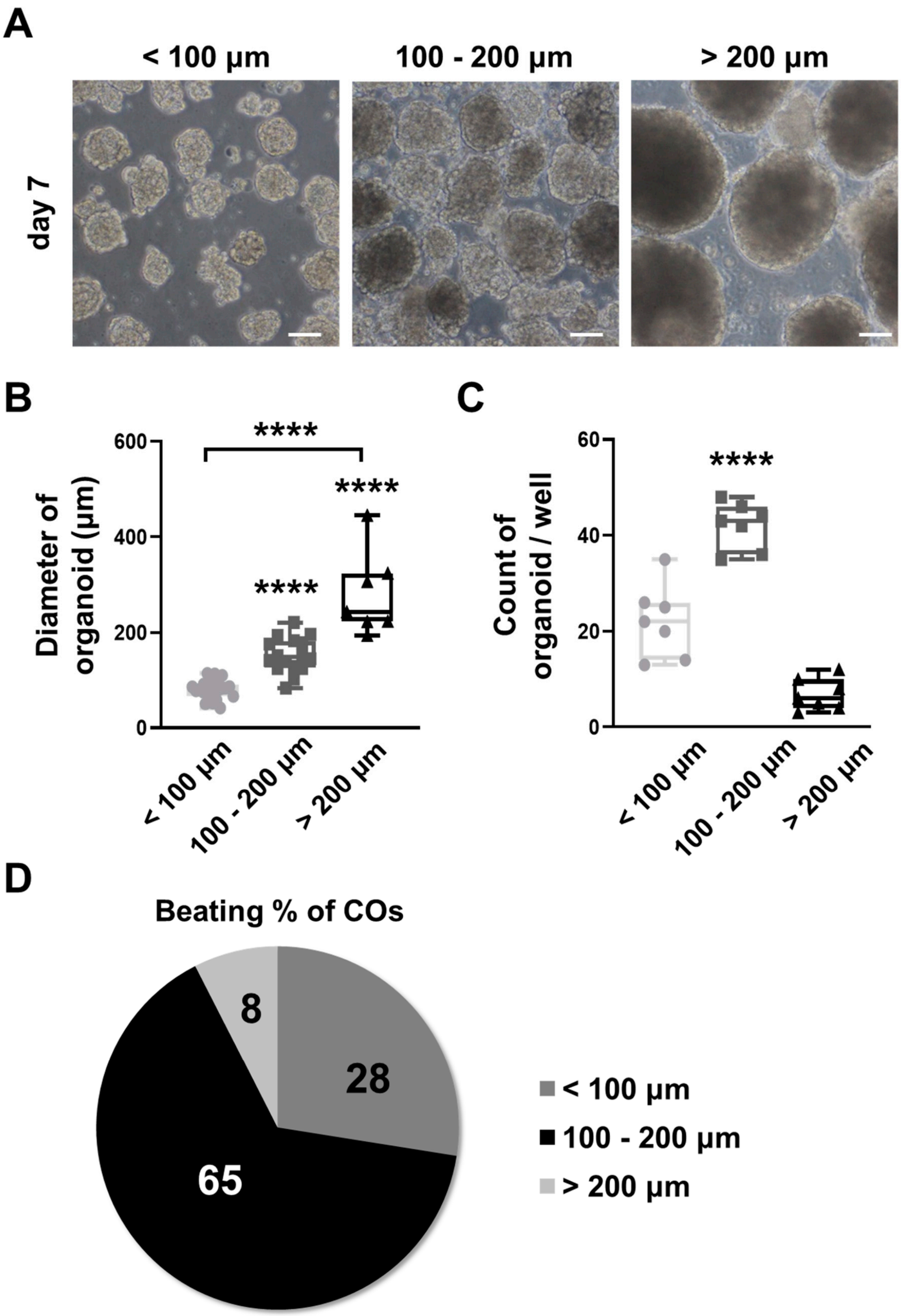
A
< 100 µm
100 - 200 µm
> 200 µm
day 7
B
Diameter of organoid (µm)
****
****
****
600
400
200
0
< 100 µm
100 - 200 µm
> 200 µm
C
Count of organoid / well
****
60
40
20
0
< 100 µm
100 - 200 µm
> 200 µm
D
Beating % of COs
8
28
65
< 100 µm
100 - 200 µm
> 200 µm

Figure S3. Generation of hESC-COs with a uniform-size using mesh filters from H9 hESCs. (A) Phase contrast images of hESC-COs after size selection with both 100- and 200-μm cell strainers on day 7. Scale bars = 100 μm. (B) The average diameters of hESC-COs divided into three groups using cell strainers on day 7: < 100 μm (n = 23), 100–200 μm (n = 16), and > 200 μm (n = 7). ****p < 0.0001. (C) The average diameters and (D) average beating percentages The average diameters of hESC-COs divided into three groups using cell strainers on day 7: < 100 μm (n = 23), 100–200 μm (n = 16), and > 200 μm (n = 7). of COs divided by size (< 100 μm, 100–200 μm, and > 200 μm) using cell strainers on day 30. n = 15. ****p < 0.0001.

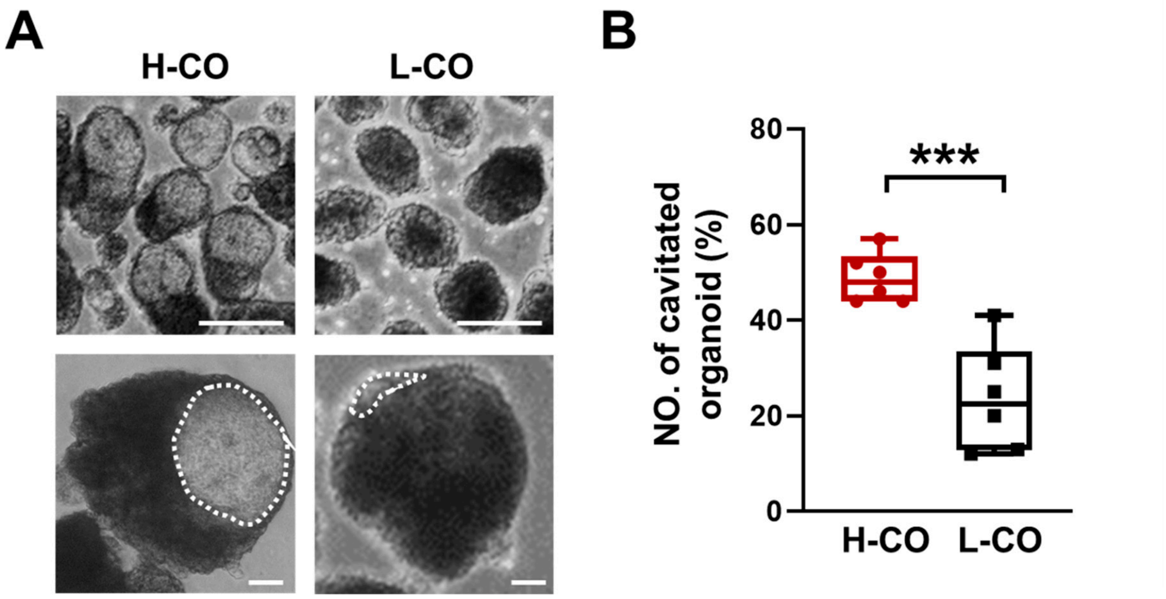

Figure S4. Increase in cavitated organoids in H-COs compared with L-COs derived from H9 hESCs. (A) Phase contrast images of cavitated H-COs and L-COs. White dashed lines indicate cavitated regions in an H-CO and L-CO. Scale bars = 100 μm. (B) Increase in the number of cavitated organoids in H-COs compared with L-COs. n = 6. ***p < 0.001.

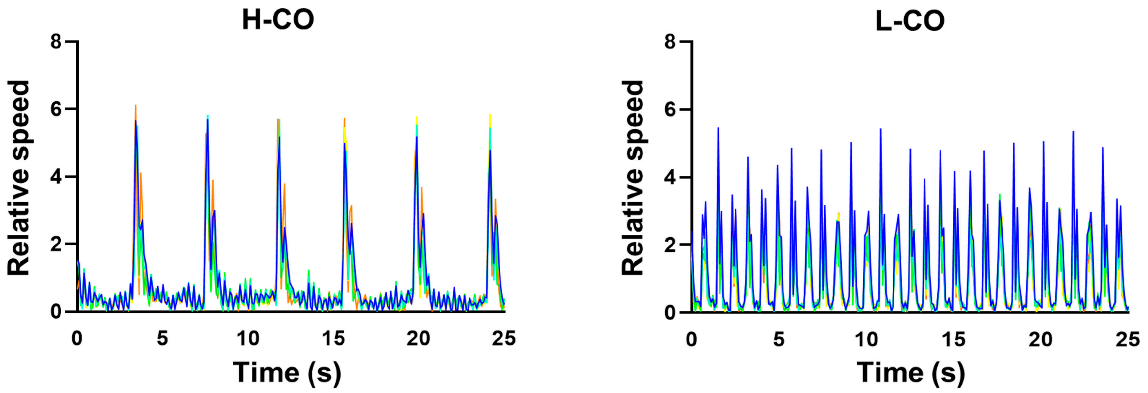

Figure S5. Representative morphologies and beat profiles in H-COs and L-COs. Beating characteristics were assessed by monitoring the light intensity of the selected regions of interest over a 25 s period in H-COs and L-COs.

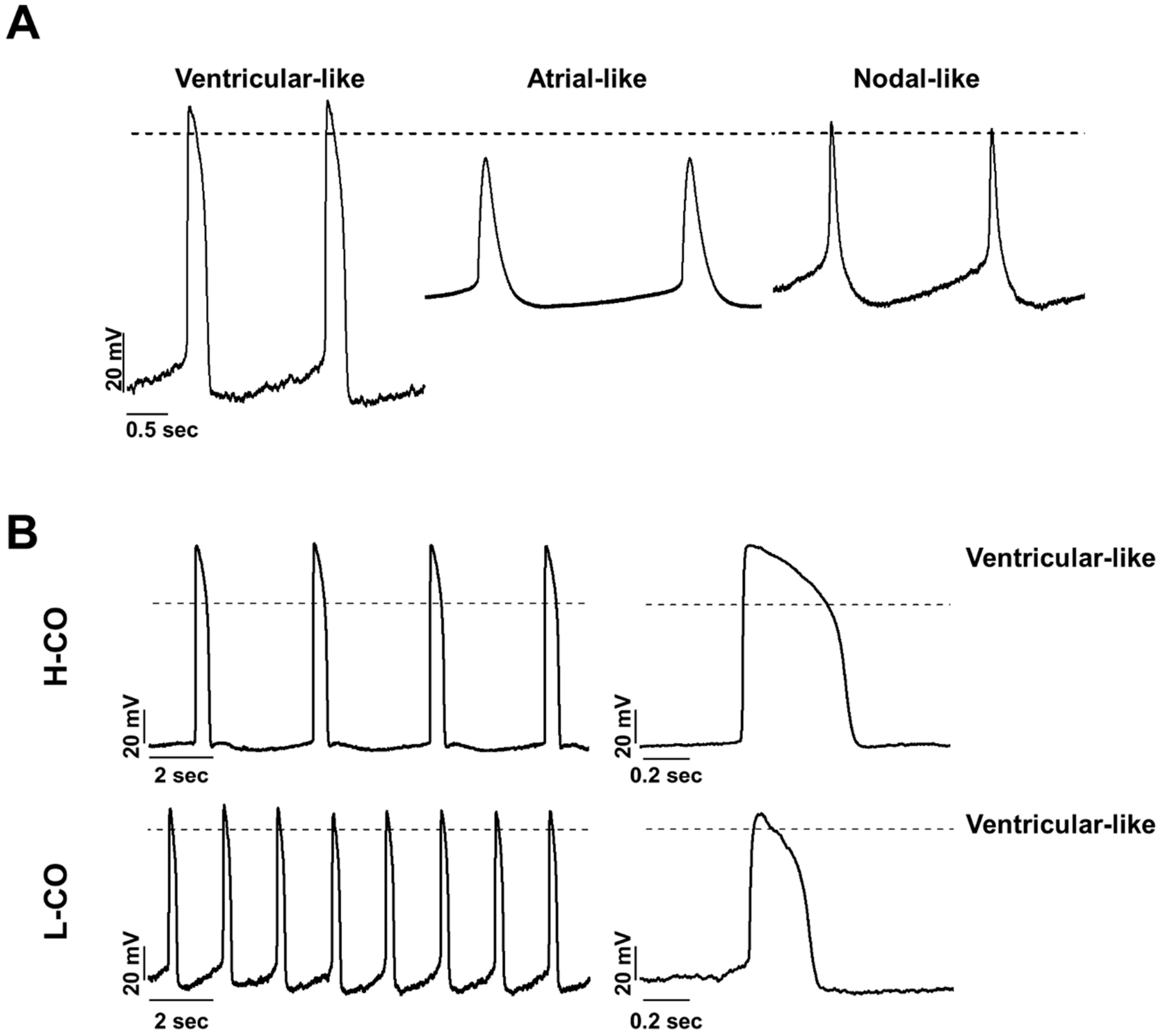

Figure S6. Prolonged APD is observed in H-COs compared with L-COs derived from H9 hESCs. (A) Representative images of the AP types in L-COs: ventricular-, atrial-, and nodal-type CMs. (B) Representative images of spontaneous APs from H-COs and L-COs.

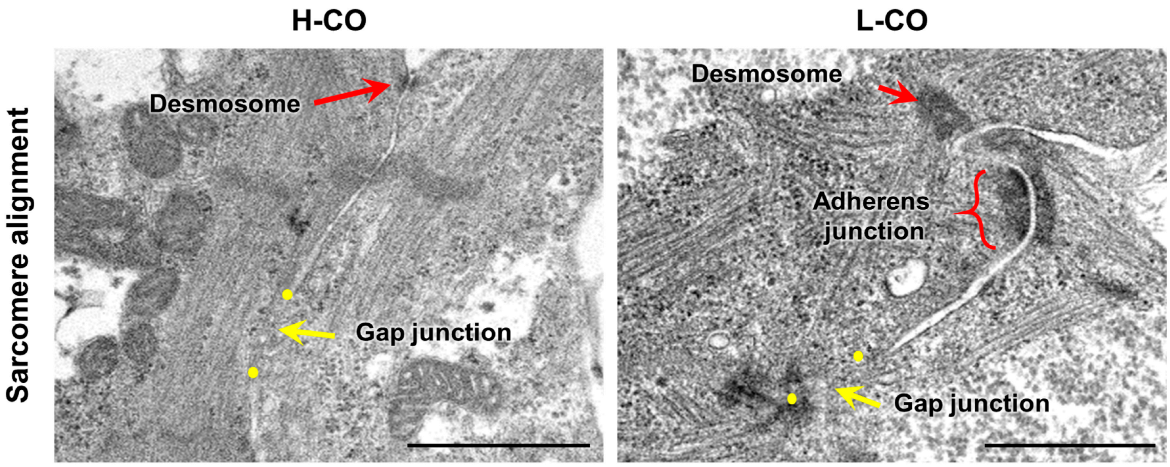

Figure S7. Sarcomeres and junctional structures between CMs are better aligned in H-COs than in L-COs derived from H9 hESCs. TEM images showing lateralized desmosomes (red arrows), adherens junctions (red brackets), and gap junctions (yellow arrows) oriented parallel to the fiber direction between two CMs in H-COs and L-COs. Scale bars = 1 μm.

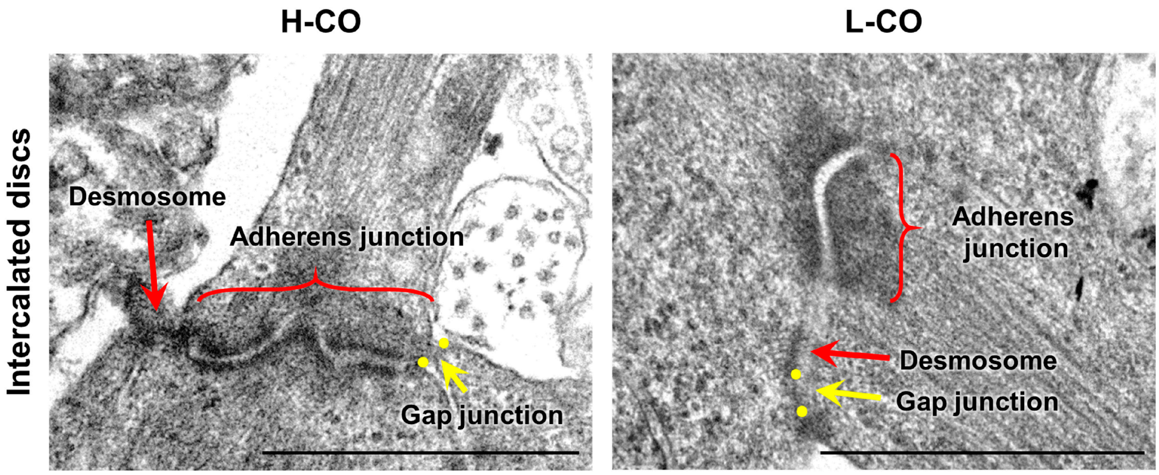

Figure S8. Intercalated discs are better organized in H-COs than in L-COs derived from H9 hESCs. Electron micrographs of parts of an intercalated disc between two CMs. TEM images show that on day 30, the intercalated discs between CMs in H-COs and L-COs derived from H9 hESCs are composed of adherens junctions (red brackets), gap junctions (yellow arrows), and desmosomes (red arrows). Scale bars = 1 μm.

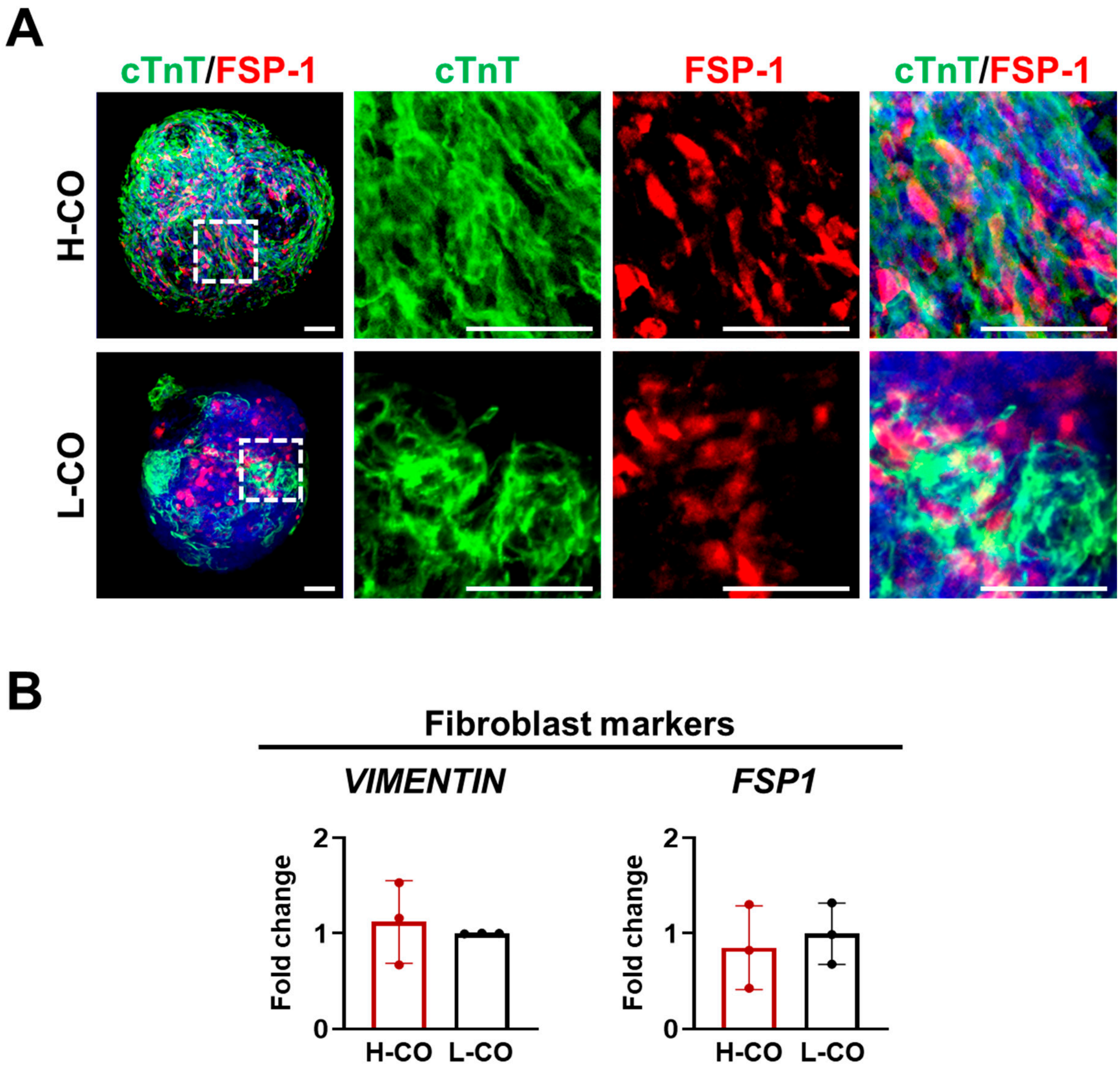

Figure S9. Fibroblasts, one of the cardiac cell types, are generated in both H-COs and L-COs derived from H9 hESCs. (A) Immunofluorescence images of a CM marker, cTnT (green), a fibroblast marker, FSP1 (red) in H-COs and L-COs. Scale bars = 20 µm. (B) qRT-PCR analysis of fibroblast makers (*VIMENTIN* and *FSP1*) in H-COs and L-COs. n = 3.

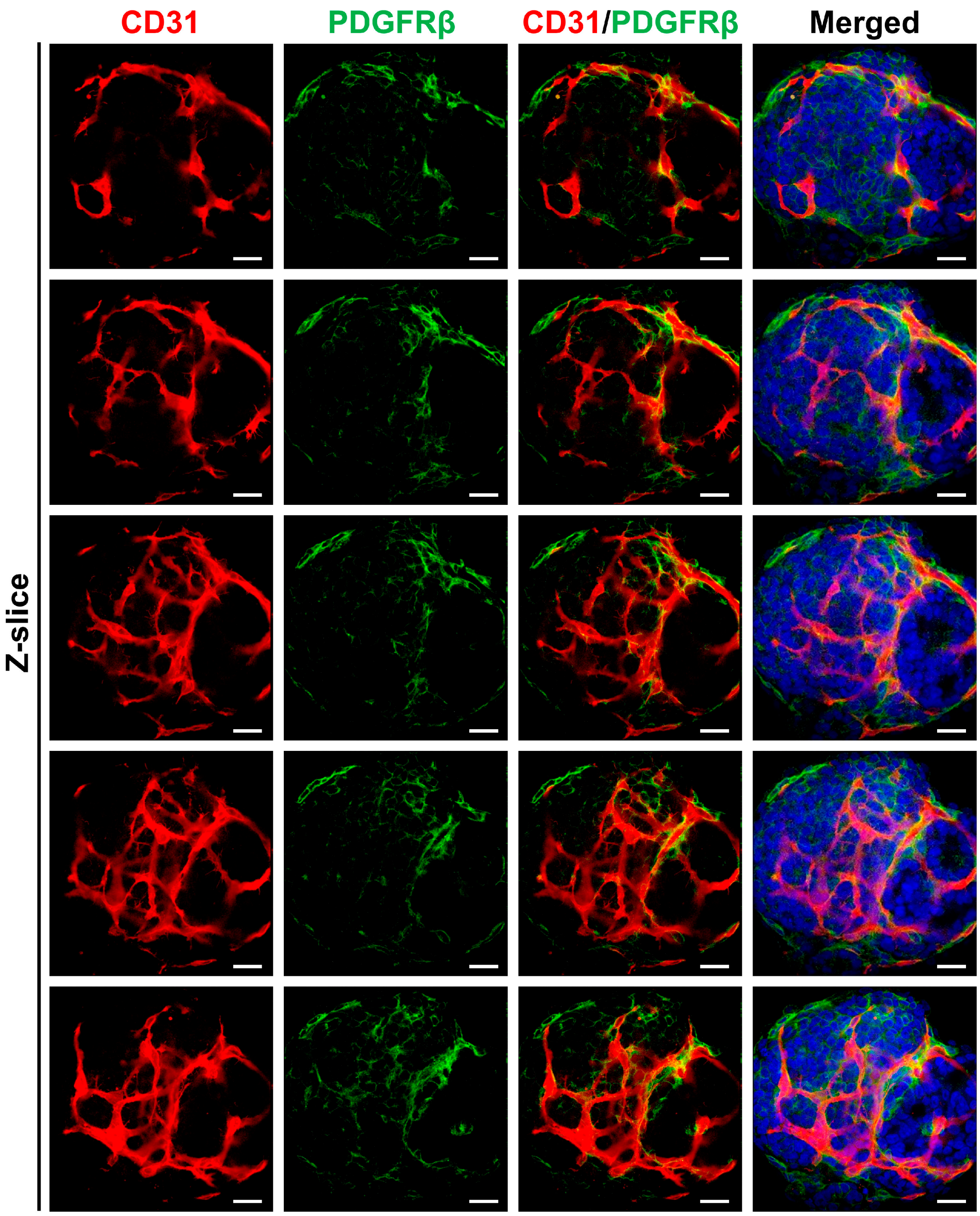

Figure S10. CD31+ ECs are well covered by PDGFRβ+ pericytes in H-COs derived from H9 hESCs. Z-stack confocal images of an EC marker, CD31 (red), and a pericyte marker, PDGFRβ (green), in H-COs. Nuclei were stained with DAPI (blue). Scale bars = 20 µm.

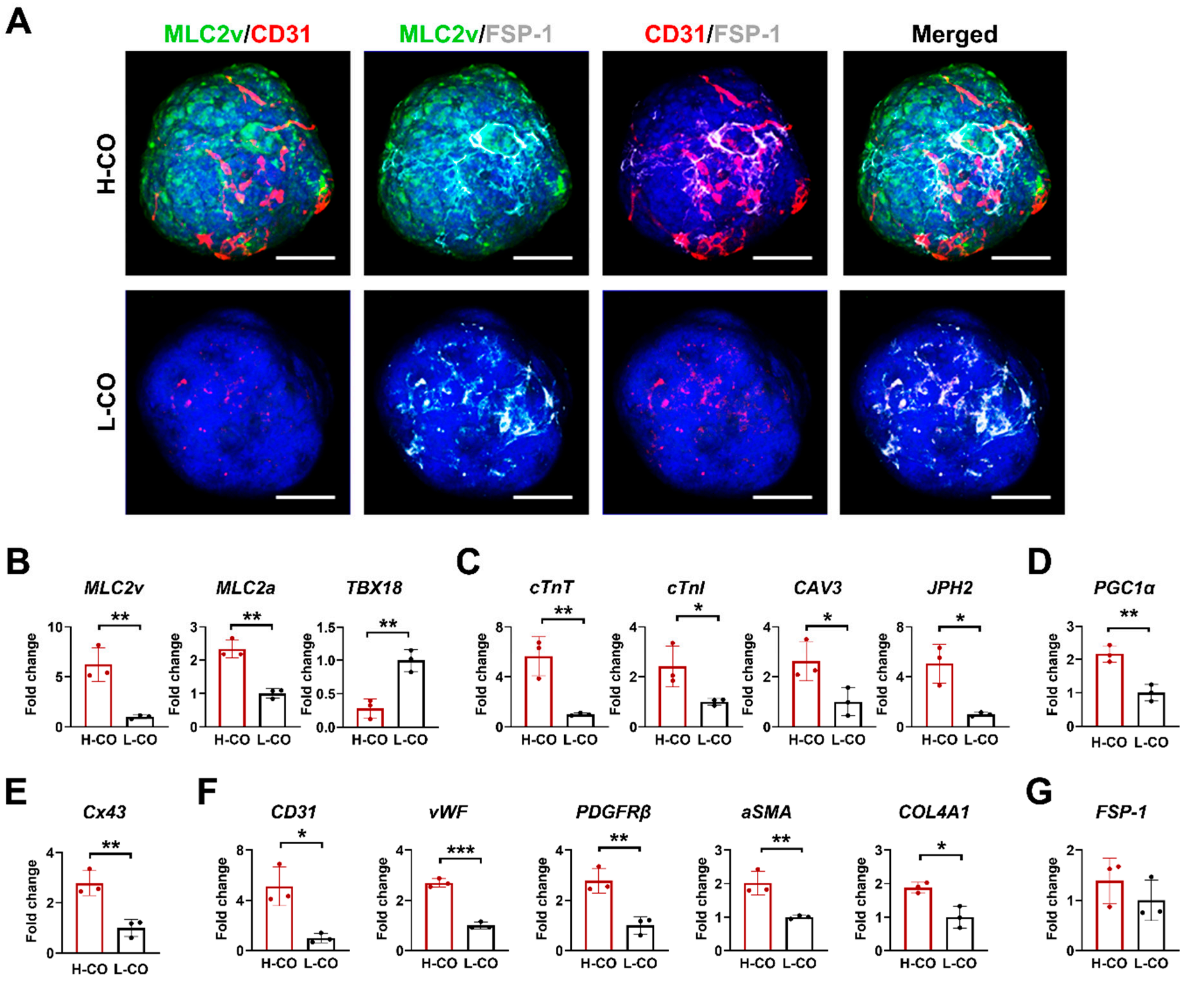

Figure S11. Enhanced expression of genes involved in CM maturation and vessel formation in H-COs compared with L-COs derived from hiPSCs. (A) Immunofluorescence images of a ventricular CM marker, MLC2v (green), an EC marker, CD31 (red), and a fibroblast marker, FSP-1 (white), in H-COs and L-COs. Scale bars = 50 μm. qRT-PCR analysis of (B) a ventricular marker (*MLC2v*), an atrial marker (*MLC2a*), and a nodal marker (*TBX18*), (C) a total CM marker (*cTnT*), a mature CM marker (*cTnI*), T-tubule markers (*CAV3* and *JPH2*), (D) a metabolic marker (*PGC1α*), (E) a gap junction marker (*Cx43*), (F) EC markers (*CD31* and *vWF*), a pericyte marker (*PDGFRβ*), an SMC marker (*αSMA*), a BM marker (*COL4A1*), and (G) a fibroblast marker (*FSP1)*

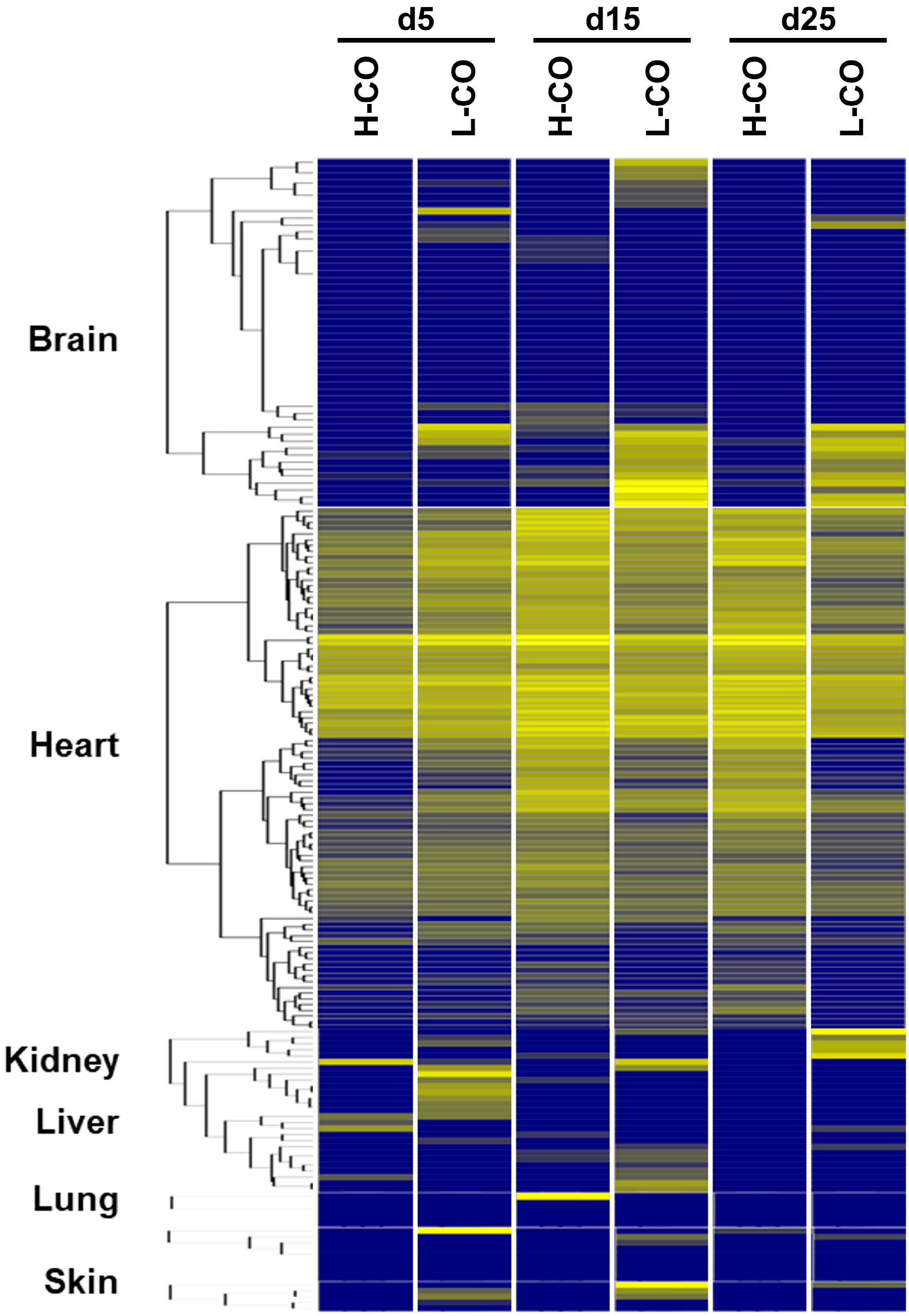

Figure S12. Heatmap of organ-specific genes obtained from RNA-Seq in H-COs and L-COs on days 5, 15, and 25 from H9 hESCs.

Table S1. Primers used for qRT-PCR

Gene	Forward primer (5'-3')	Reverse primer (5'-3')	Product (bp)
AFP	TGAACGGCATGAACACGTA	GCCCACGTACGACGACAT	101
CALPONIN1	TAAGAGAAGGGCGGAACATC	GCTTGGGGTCGTAGAGGTG	108
CAV3	GAGGCCCAGATCGTCAAG	TCACGTCTTCAAAATCCACCT	106
CD117	ATGGCATGCTCCAATGTGT	GGCAGTACAGAAGCAGAGCA	100
CD31	GAGTCCTGCTGACCCTTCTG	TCAGGTTCTTCCCATTTTGC	120
COL4A1	CCAGGACCAAGTGGAAGAGA	GGGCTGACATTCCACAATTC	99
COL4A2	GGGGGAACCAGGAATAAGAG	ACCACTCAAGCCAGGGTAAC	91
CPT1B	TGTGAGTGACTGGTGGGAAG	TTGATGAGCACAAGGTCCAT	96
cTnI	GCAGATGCCATGATGCAG	CACCTCCCGGTTTTCCTT	114
cTnT	GTCGGCAGCTGCTGTTCT	TCCTCTCTCCAGTCCTCCTCT	124
CXCR4	CCTGCCTGGTATTGTCATCC	AGGATGACTGTGGTCTTGAGG	105
EFNB2	GTGTGCCAGACAAGAGCCATGAAG	GGGACTTGTTGTCGAACTTCTTCC	117
eNOS	GACTGAAGGCTGGCATCTG	CCATGTTACTGTGCGTCCA	107
EPHB4	GTGGTCATTGTGGTCGCAGTTCTC	CCTCACAGCCTCATTAGGGTCTTC	129
FSP1	TTCAGTCACAATGCCGTGATT	TTCAGTCACAATGCCGTGATT	105
GAPDH	GAGTCCACTGGCGTCTTCAC	TTCACACCCATGACGAACAT	119
JPH2	GAGGTGGAGGTGGAAGAGG	TCAGGTCAGGAGGTGAACAA	99
KCNA4	CCCACCCAGGATCATTCTT	TCATGCAGAAGAAGCACTTCAC	130
KCNH2	TTCAGTCACAATGCCGTGATT	GCTTTTCCGAAGATTGCCCA	142
KCNJ2	CAACCTGGGCGACCAGATAG	GGTGTTGGGAGAGACGTTGC	78
MESP1	CCGAGTCCTGGATGCTCTC	AGTCTGGGGACGAGACGAG	96
MLC2a	GGGTGGTGAACAAGGATGAG	GTGTCAGGGCGAACATCTG	93
MLC2v	GCAGGCGGAGAGGTTTTC	AGTTGCCAGTCACGTCAGG	74
NANOG	GATTTGTGGGCCTGAAGAAA	AAGTGGGTTGTTTGCCTTTG	155
OCT4	AGTGAGAGGCAACCTGGAGA	GCCGGTTACAGAACCACACT	125
OTX2	GGGTATGGACTTGCTGCAC	AGTGCTTCCAGCACATCTAGC	108
PDGFRβ	GTGCTGGGAAGAGAAGTTTGA	TCATCCACCTGCTGGTACTTC	108
PGC1A	CATCAGCAATGGATGAGACC	CCAACCAGAGCAGCACACT	94
PROX1	GCTCCAATATGCTGAAGACC	ATCGTTGATGGCTTGACGTG	140
T	GCTGTGACAGGTACCCAACC	CATGCAGGTGAGTTGTCAGAA	106
TBX18	CCTGGAATTCCCAAGCAAG	GAGGAGCCAGACAAAAGGTG	101
TFAM	GTTTCTCCGAAGCATGTGG	AGATGAAAACCACCTCGGTAAA	127
TIE2	GGACCTGAATGCAACCATCT	TTCACAAGCCTTCTCACACG	121
VIMENTIN	TACAGGAAGCTGCTGGAAGG	ACCAGAGGGAGTGAATCCAG	104
vWF	TAAGTCTGAAGTAGAGGTGG	AGAGCAGCAGGAGCACTGGT	109
ZIC2	ATCCACAAAAGGACGCACAC	GTCACAGCCCTCAAACTCG	60
αSMA	ATCCCCGGGACTAAGACG	CAAAGCCGGCCTTACAGAG	113

Table S2. Antibodies for immunostaining

Antibody	Company	Product number	Concentration
CAV3	Abcam	ab2912	1:500
CD31	DAKO	M0823	1:200
cTnT	Thermo Fisher Scientific	MS295	1:400
Cx43	Abcam	ab11370	1:500
FOXA2	Abcam	ab108422	1:250
FSP1	Merck Millipore	07-2274	1:500
JPH2	Thermo Fisher Scientific	40-5300	1:500
MIXL1	Novus Biologicals	NBP2-55175	1:500
MLC2a	Synaptic Systems	#311011/56F5	1:400
MLC2v	ProteinTech	#PTG10906-1-AP	1:200
NESTIN	R&D Systems	MAB1259	1:500
OCT4	SantaCruz	sc-5279	1:500
PDGFRβ	Abcam	ab32570	1:500
T	Novus Biologicals	NBP2-24676	1:500
TBX18	R&D Systems	MAB63371	1:400
VEGFR2	Abcam	ab2349	1:100
vWF	BD Biosciences	555849	1:500
αSMA	Sigma-Aldrich	C6198	1:400

Table S3. Antibodies for western blotting

Antibody	Company	Product number	Concentration
CAV3	Abcam	ab2912	1:500
CD31	DAKO	M0823	1:50
Cofilin	Abcam	ab42824	1:1000
pCofilin	Cell signaling technology	3313	1:1000
COL1A	Cell signaling technology	66948	1:1000
CPT1β	Proteintech	22170-1-AP	1:1000
cTnT	Thermo Fisher Scientific	MS295	1:1000
Cx43	Abcam	ab11370	1:1000
FAK	Cell signaling technology	13009	1:1000
pFAK	Cell signaling technology	8556	1:1000
eNOS	Cell signaling technology	9572	1:1000
peNOS	Cell signaling technology	9571S	1:1000
FN1	Cell signaling technology	26836	1:1000
GAPDH	Sigma–Aldrich	G8795	1:20000
HIF-2α	Cell signaling technology	7096	1:1000
ITGA5	Cell signaling technology	4705	1:1000
ITGAV	Cell signaling technology	4711	1:1000
ITGB1	Cell signaling technology	9699	1:1000
ITGB3	Cell signaling technology	13166	1:1000
ITGB4	Cell signaling technology	14803	1:1000
ITGB5	Cell signaling technology	3629	1:1000
JPH2	Thermo Fisher Scientific	40-5300	1:1000
Laminin	Invitrogen	PA1-16730	1:1000
LEFTY	Abcam	ab22569	1:1000
LIMK1	Cell signaling technology	3843	1:1000
pLIMK1	Cell signaling technology	3841	1:1000
MLC2	Cell signaling technology	8505	1:1000
pMLC2	Cell signaling technology	3671	1:1000
MLC2a	Synaptic Systems	#311011/56F5	1:1000
MLC2v	ProteinTech	#PTG10906-1-AP	1:1000
NODAL	Abcam	ab55676	1:1000
PDGFRα	Cell signaling technology	3164	1:1000
pPDGFRα	Cell signaling technology	2992	1:1000
PDGFRβ	Abcam	ab32570	1:1000
pPDGFRβ	Cell signaling technology	2227S	1:1000
PGC1α	Novus Biologicals	NBP104676	1:1000
PITX2	Abcam	ab32832	1:1000

RAC1	Cell signaling technology	4651	1:1000
pRAC1	Cell signaling technology	2461	1:1000
ROCK1	Abcam	ab45171	1:1000
ROCK2	Abcam	ab71598	1:1000
SMAD1	Cell signaling technology	6944	1:1000
pSMAD1/5	Cell signaling technology	9516	1:1000
pSMAD2	Cell signaling technology	3108	1:1000
SMAD3	Cell signaling technology	9523	1:1000
pSMAD3	Cell signaling technology	9520	1:1000
SMAD5	Cell signaling technology	9517	1:1000
TBX18	R&D	MAB63371	1:1000
TFAM	Abcam	ab131607	1:1000
VEGFR2	Cell signaling technology	2479S	1:1000
pVEGFR2	Cell signaling technology	2478S	1:1000
ZO-1	Thermo Fisher Scientific	33-9100	1:1000
vWF	BD Biosciences	555849	1:500
αSMA	Sigma-Aldrich	C6198	1:400

Legends to Supplementary Videos

Legend to Supplementary Video S1: Representative morphologies and magnitudes of motion velocity in H-COs from H9 hESCs.

Legend to Supplementary Video S2: Fluorescence signals recorded from expressing CD31 (red) and cTnT (green) in H-CO are shown from H9 hESCs.

Legend to Supplementary Video S3: Immunofluorescence signals recorded the formation of lumen consisting of CD31+ ECs in H-COs are shown from H9 hESCs.
