# Supplementary figures and images for: The Activation of the LIMK/Cofilin Signaling Pathway via Extracellular Matrix–Integrin Interactions Is Critical for the Generation of Mature and Vascularized Cardiac Organoids
Source: Cells. 2023 Aug 9;12(16):2029. doi: 10.3390/cells12162029 (PMC10453200; doi:10.3390/cells12162029)

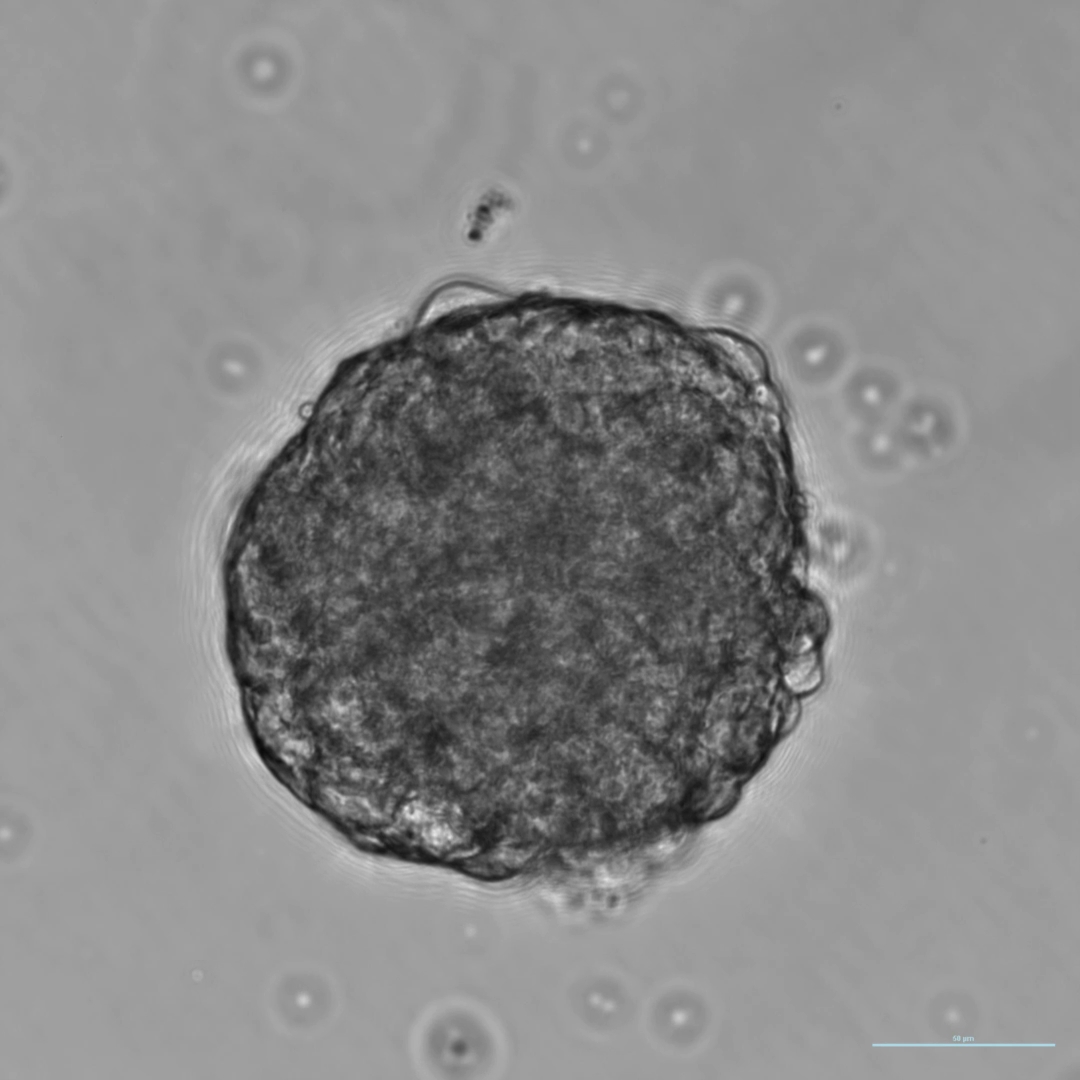

Supplement: Supplementary file 1 [file cells-12-02029-s001.zip › [Video] Supplementary Video S1_Chapter.jpg]

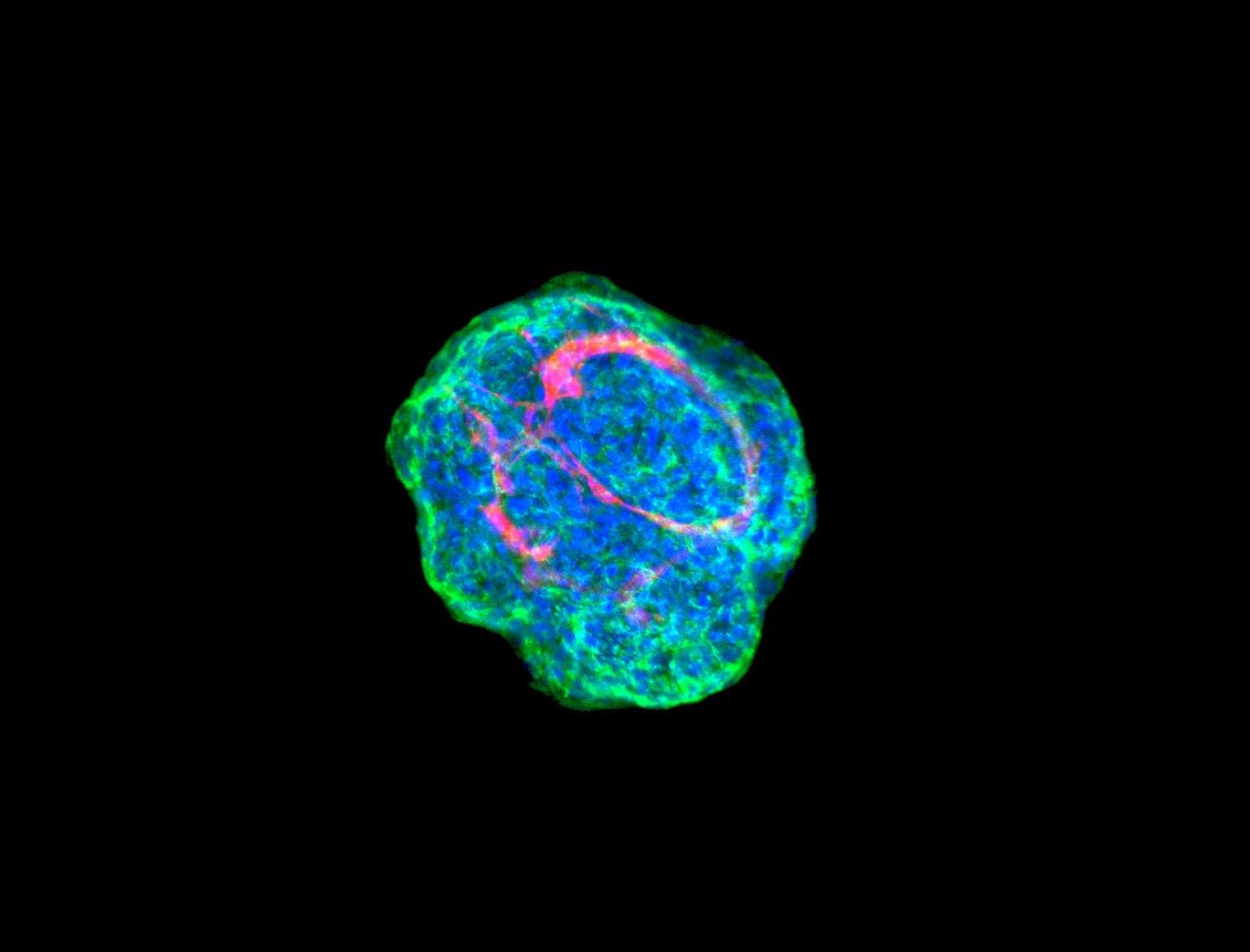

Supplement: Supplementary file 1 [file cells-12-02029-s001.zip › [Video] Supplementary Video S2_Chapter.jpg]

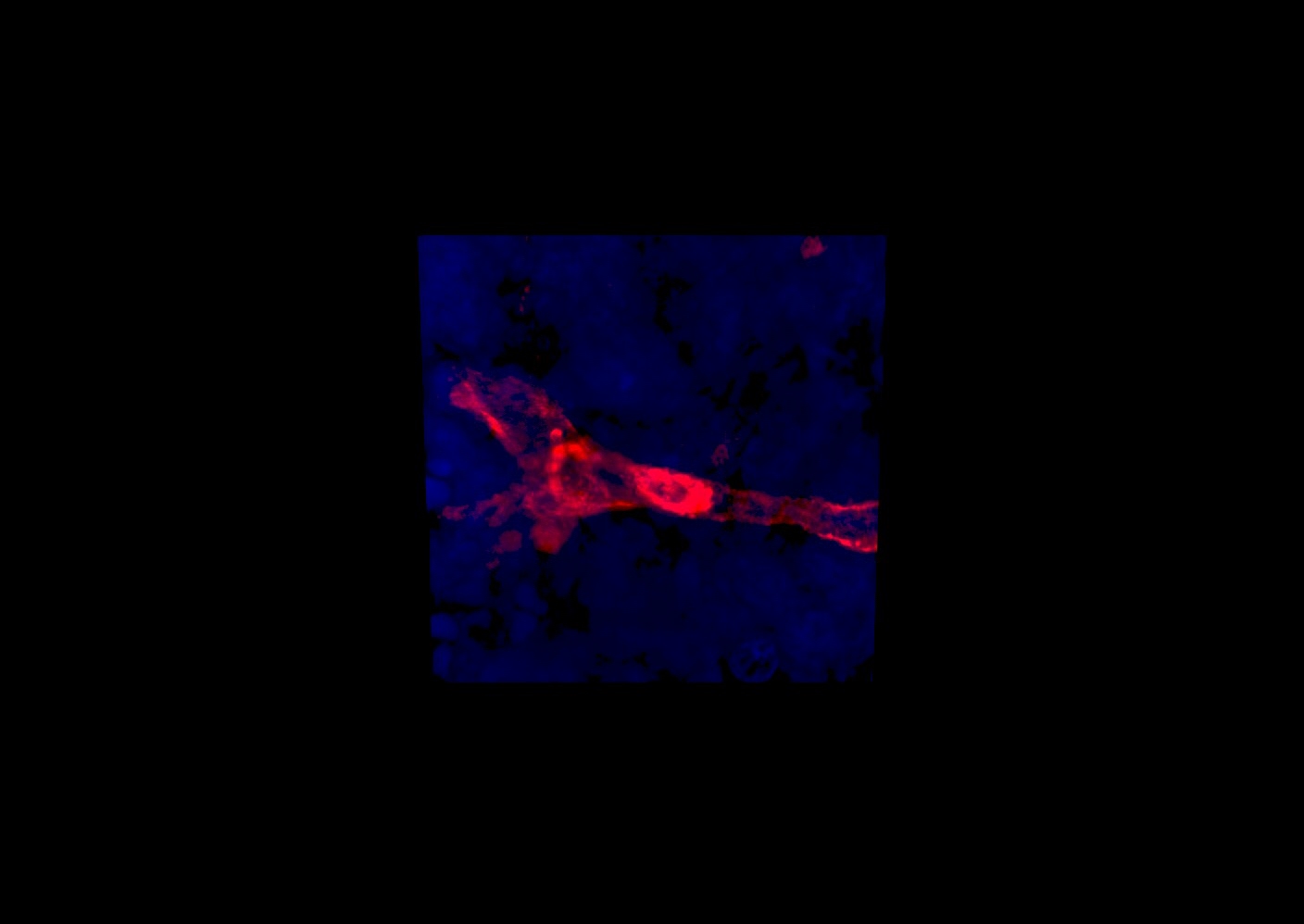

Supplement: Supplementary file 1 [file cells-12-02029-s001.zip › [Video] Supplementary Video S3_Chapter.jpg]
